# A Brain Region-Specific Expression Profile for Genes Within Large Introgression Deserts and Under Positive Selection in *Homo sapiens*

**DOI:** 10.3389/fcell.2022.824740

**Published:** 2022-04-26

**Authors:** Raül Buisan, Juan Moriano, Alejandro Andirkó, Cedric Boeckx

**Affiliations:** ^1^ Universitat de Barcelona, Barcelona, Spain; ^2^ Universitat de Barcelona Institute of Complex Systems, Barcelona, Spain; ^3^ Catalan Institute for Research and Advanced Studies (ICREA), Barcelona, Spain

**Keywords:** Homo sapiens, deserts of introgression, positive selection, cerebellum, striatum, thalamus, gene expression

## Abstract

Analyses of ancient DNA from extinct hominins have provided unique insights into the complex evolutionary history of *Homo sapiens*, intricately related to that of the Neanderthals and the Denisovans as revealed by several instances of admixture events. These analyses have also allowed the identification of introgression deserts: genomic regions in our species that are depleted of “archaic” haplotypes. The presence of genes like *FOXP2* in these deserts has been taken to be suggestive of brain-related functional differences between *Homo* species. Here, we seek a deeper characterization of these regions and the specific expression trajectories of genes within them, taking into account signals of positive selection in our lineage. Analyzing publicly available transcriptomic data from the human brain at different developmental stages, we found that structures outside the cerebral neocortex, in particular the cerebellum, the striatum and the mediodorsal nucleus of the thalamus show the most divergent transcriptomic profiles when considering genes within large introgression deserts and under positive selection.

## 1 Introduction

The availability of high-coverage genomes from our closest extinct relatives, the Neanderthals and Denisovans, constitutes a significant advance in the range of questions one can ask about the deep history of our species (Meyer et al., 2012; Prüfer et al., 2014; Prüfer et al., 2017; Mafessoni et al., 2020). One of the main themes emerging from this progress is interbreeding. In recent years, a fairly large number of admixture events between Neanderthals, Denisovans and Sapiens populations have been postulated. A recent review (Bergström et al., 2021) considers that at least four such events are supported by strong evidence.

While it is important to ask whether our species benefited from these admixture events (so-called adaptive introgression, where alleles inherited from other hominins rose to high frequency as a result of positive selection after gene flow), it is also worth examining regions of the genomes that are depleted of alleles resulting from gene flow from other hominins (Sankararaman et al., 2016; Vernot et al., 2016; Chen et al., 2020; Skov et al., 2020; Rinker et al., 2020). Such regions are called introgression deserts (sometimes also “genomic islands of divergence/speciation” (Wang et al., 2020) and have now been identified in a range of species (Fontsere et al., 2019).

There are multiple reasons why genetic differences that arose after the divergence of populations may not be well tolerated (Wolf and Akey, 2018): there could be negative selection on “archaic” variants (deleterious changes on the “archaic” lineage), or positive selection on human-specific variants (adaptive changes on the human lineage), or it may be due to drift. It is reasonable to expect, and indeed has been shown, that the X chromosome constitutes such a desertic region [not only in our species (Kuhlwilm et al., 2019; Martin and Jiggins, 2017)]. This could be due to repeated selective sweeps on this chromosome: genes involved in reproduction on this chromosome might act as strong reproductive barriers between populations (Fontsere et al., 2019).

In the case of modern humans, other genomic regions are devoid of Neanderthal and Denisovan introgression, for reasons that are perhaps less obvious, and therefore worth investigating further. A recent study (Chen et al., 2020) identifies four large deserts depleted of Neanderthal introgression, partially overlapping with a previous independent study (Vernot et al., 2016). As pointed out in (Kuhlwilm, 2018; Wolf and Akey, 2018), since it is likely that there were several different pulses of gene flow between us and our closest relatives (Iasi et al., 2021), the depletion observed in these four regions must have been reinforced repeatedly, and given the size of the deserts, it is reasonable that the “archaic” haplotype was purged within a short time after the gene flow event, as predicted by mathematical modeling on whole-genome simulations (Veller et al., 2021), and as evidenced in the analysis of genome-wide data from the earliest Late Pleistocene modern humans known to have been recovered in Europe (Hajdinjak et al., 2021).

The presence of *FOXP2*, a gene known for its role in language (Lai et al., 2001; Fisher, 2019), in one of these large deserts has attracted attention (Kuhlwilm, 2018), as it raises the possibility that the incompatibility between *Homo sapiens* and other hominin in such persistent introgression deserts may point to (subtle, but real) cognitive/behavioral differences. Indeed, the presence in such deserts of not only *FOXP2* but also other genes like *ROBO1*, *ROBO2*, and *EPHA3*, all independently associated with language traits (St Pourcain et al., 2014; Wang et al., 2015; Eising et al., 2021; Mekki et al., 2022), together with an earlier observation in Vernot et al. (2016) that genes within large deserts are significantly enriched in the developing cerebral cortex and in the adult striatum, suggest a possible point of entry into some of the most distinctive aspects of the human condition (Pääbo, 2014). Such considerations, combined with independent evidence that introgressed Neanderthal alleles show significant downregulation in brain regions (McCoy et al., 2017), motivated us to focus on the brain in this study.

**FIGURE 1:**
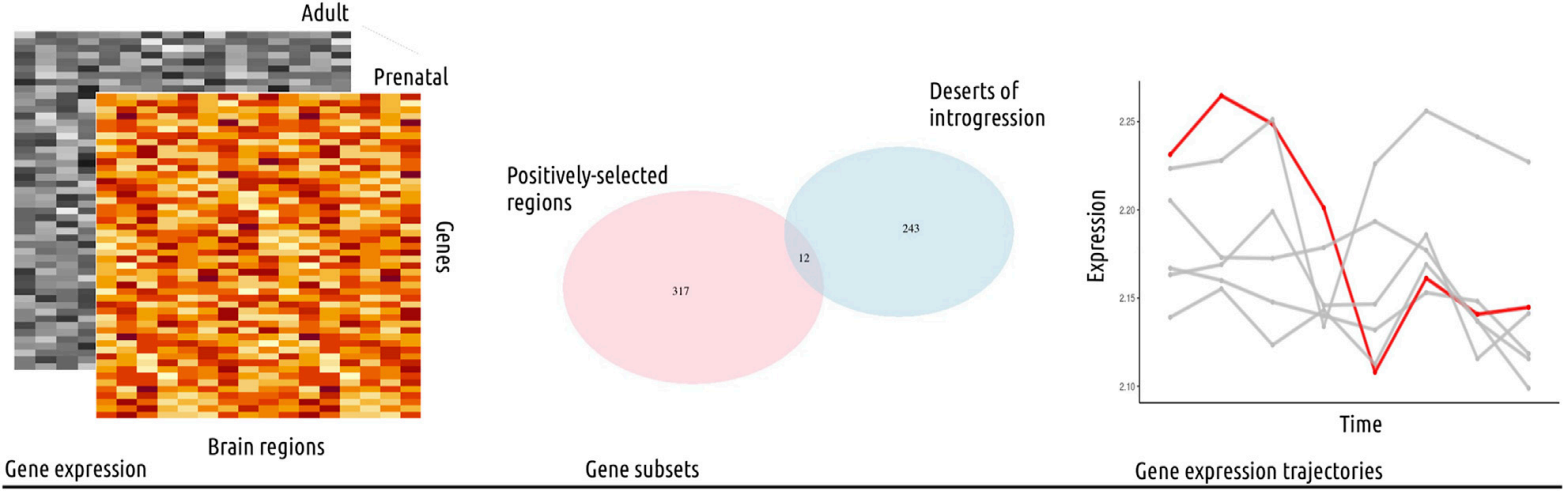
Study outline. In this study, we used publicly available gene expression data from the psychENCODE project (Li et al., 2018), covering sixteen brain regions and eight developmental stages, from prenatal to adult (left panel). The data was analyzed following standard procedures and the normalized, log-transformed gene expression values were then used for further analysis. We focused on gene subsets from genomic regions with evolutionary-relevant properties (middle panel): 1) positively-selected regions and 2) large deserts of introgression (see also Table 1). We investigated the expression trajectories of genes fulfilling both of these conditions (twelve genes in total) across brain regions, and proceeded to compare their gene expression values to control genes from length and gene density-matched random regions, as well as to other subsets of genes. Specifically, we performed a Principal Component Analysis and calculated the Euclidean distance between each data point (gene expression value for a subset of genes in a particular brain region) per developmental stage to identify statistically significant differences (Wilcoxon rank sum test with Bonferroni correction) among brain regions. This analysis was complemented with a segmented regression model for a single-gene analysis of gene expression dynamics (right panel). Collectively, we found the most significant differences for the cerebellum, the striatum and the mediodorsal nucleus of the thalamus.

Specifically, we focused on the four largest genomic regions that resisted “archaic” introgression reported in (Chen et al., 2020), jointly with the most comprehensive catalog to date of signals of positive selection in our lineage (Peyrégne et al., 2017) (see Table 1), a combination that, to our knowledge, has not been previously studied in detail. Here, we tested if the genes that fulfill these two conditions (falling within large deserts of introgression and being under positive selection) follow particular (brain-region) expression trajectories that significantly deviate from that of other subsets of genes with evolutionary relevance or from control genomic regions. We characterized the gene expression dynamics (including genes falling within either deserts of introgression or positively-selection regions alone) by analyzing transcriptomic data from several brain regions encompassing multiple developmental stages from prenatal to adulthood. This dataset allows for greater resolution than the Allen Brain Atlas data used in (Vernot et al., 2016), especially at early stages of development (see Figure 1). Three of the brain regions under study showed marked transcriptomic divergence (i.e., a statistically significant difference when compared to all other regions, based on the Principal Component Analysis-derived Euclidean distances): the cerebellum, the striatum and the thalamus. Among the genes at the intersection of regions under positive selection and large deserts of introgression, we found *CADPS2*, *ROBO2*, or *SYT6*, involved in neurotrophin release, axon guidance and neuronal proliferation, and known to be expressed in the brain regions our analysis highlights.

**TABLE 1:** Genomic coordinates used in this study. Large deserts were retrieved from (Chen et al., 2020), and positively-selected regions from (Peyrégne et al., 2017) (see Section 4). The circos plot on the right shows the distribution of our regions of interest: Blue bloxes: deserts of introgression; Red lines: positively-selected regions within deserts of introgression. Colored regions within the brain represent structures that figure prominently in this study.

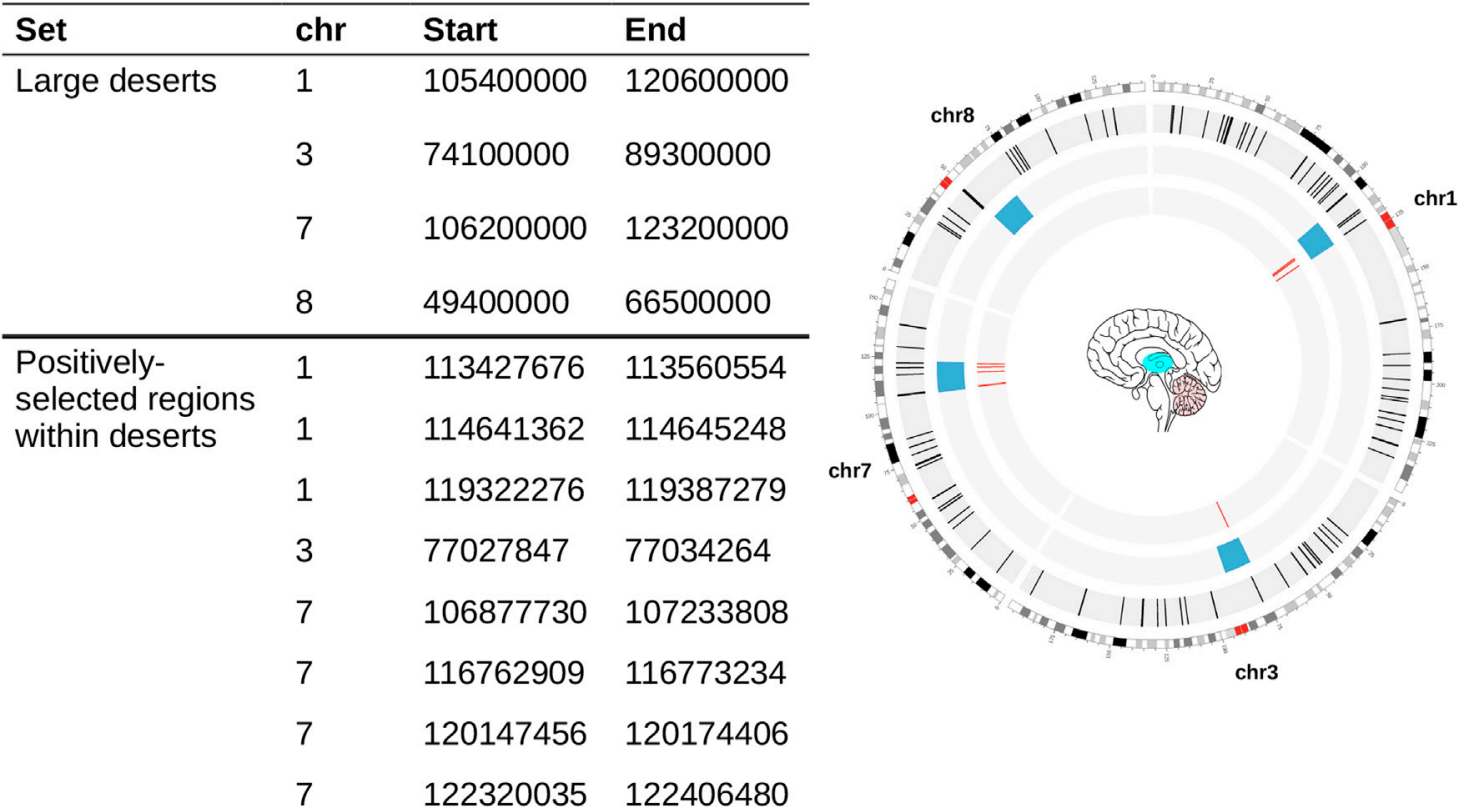

## 2 Results

### 2.1 Genes in Large Deserts of Introgression Have Different Expression Levels Relative to the Rest of the Genome

We set out to understand whether the mean expression of genes in large deserts of introgression (Chen et al., 2020) and the positively selected regions within them (extracted from (Peyrégne et al., 2017)) is significantly different compared to the rest of the genome, using publicly available transcriptomic data from the human brain (Li et al., 2018). To this end, we selected random regions of the genome (*n* = 1,000), excluding the large deserts, of the same average length (i.e., 15 million base-pairs), with a possible deviation of 1 million base-pairs to account for the length variability between different deserts of introgression. To avoid genomic regions with low genetic density that might skew the results, the randomized areas were required to hold at least as many genes (265) as the desertic regions reported in (Chen et al., 2020).

The mean expression of genes lying in random regions of the genome was summarized for each brain structure (and log2-transformed). A repeated-measures two-way anova shows that the mean expression of both sets of these regions is significantly different from the rest of the genome (*p* < 0.01 for both sets). A post-hoc pairwise anova (with Bonferroni correction) shows the difference between a gene expression value in a brain region as derived from the control set and that obtained from the genes in our two sets of interest is significant for most structures. An outlier’s Grubbs test shows that the structures with the highest and lowest mean gene expression values in large deserts of introgression and the positively-selected windows within them fall inside the expected range of variability given the data (*p* > 0.01).

### 2.2 The Cerebellar Cortex, the Striatum and the Thalamus Show Divergent Transcriptomic Profiles When Considering Genes Within Large Deserts of Introgression and Under Positive Selection

We then investigated the temporal progression of the expression of genes within large deserts of introgression and putative positively-selected regions analyzing RNA-seq data of different human brain regions at different developmental stages (Li et al., 2018). We found that the median expression of genes within large deserts and positively-selected regions is higher than those present in deserts alone, the former peaking at prenatal stages in neocortical areas and decreasing later on. Outside the cerebral neocortex, this pronounced prenatal peak is not observed and, specifically for the cerebellar cortex, the expression profile of these genes increases before birth and reaches the highest median expression from childhood to adulthood in comparison to the rest of structures (see Supplementary Figure S1).

**FIGURE 2:**
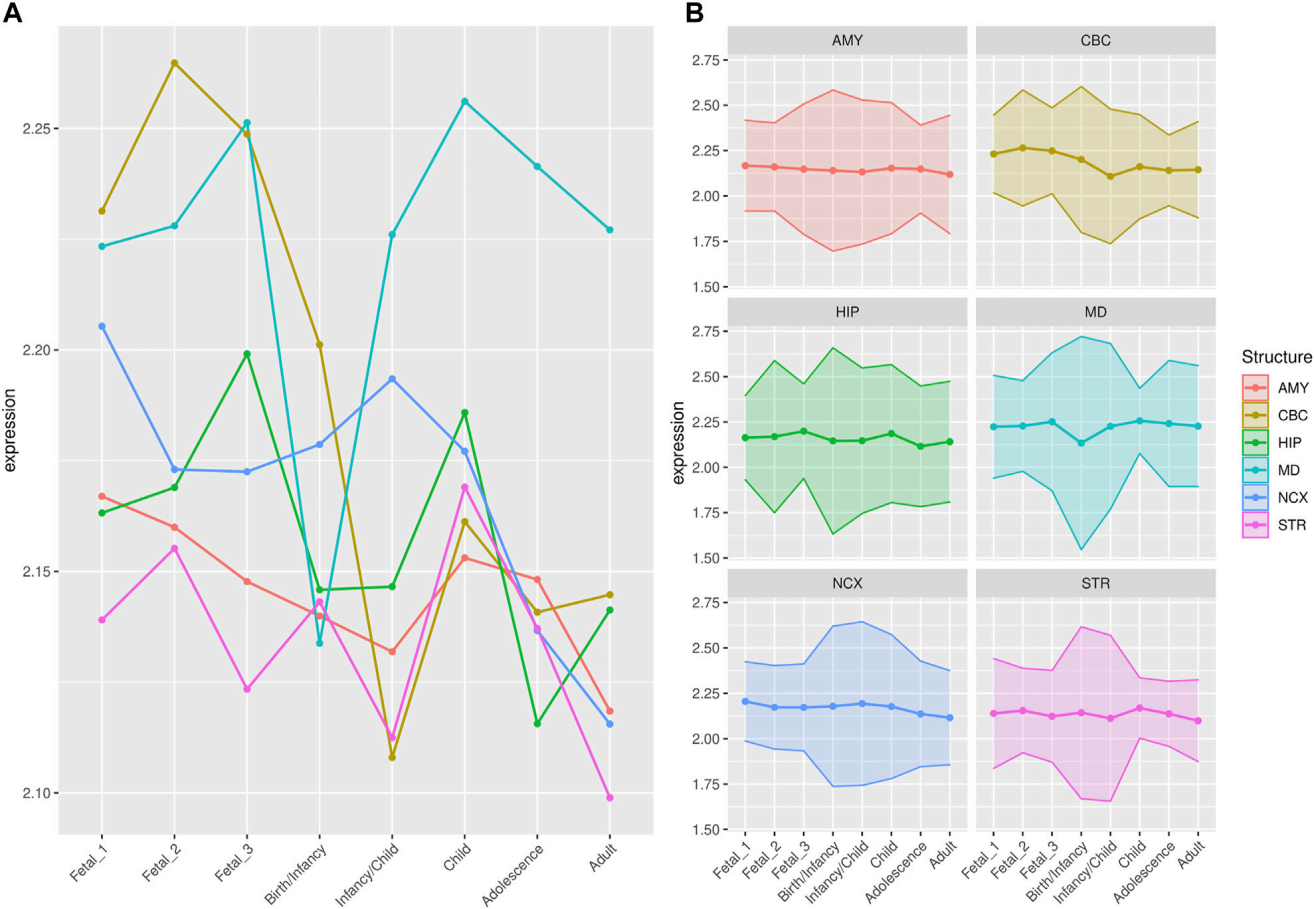
Median expression of genes within large deserts. **(A)** Comparison of median of gene expression across developmental stages between structures and **(B)** Standard deviation per structure, for genes (*n* = 255) within the four large deserts of introgression. The cerebellar cortex, prenatally, and the mediodorsal nucleus of the thalamus prenatally and postnatally present the highest expression. The median expression profile of genes within deserts, per chromosome, is shown in Supplementary Figure S12. *Structures:* AMY, amygdala; CBC, cerebellar cortex; HIP, hippocampus; MD, mediodorsal nucleus of thalamus; NCX, neocortex; STR, striatum. *Stages:* Fetal 1: 12-13 post conception weeks (PCWs); Fetal 2: 16-18 PCW; Fetal 3: 19-22 PCW; Birth—Infancy: 35-37 PCW and 0–0.3 years; Infancy—Child: 0.5–2.5 years; Childhood: 2.8–10.7 years, Adolescence: 13–19 years; Adulthood: 21–64 years [as in (Li et al., 2018)].

**FIGURE 3:**
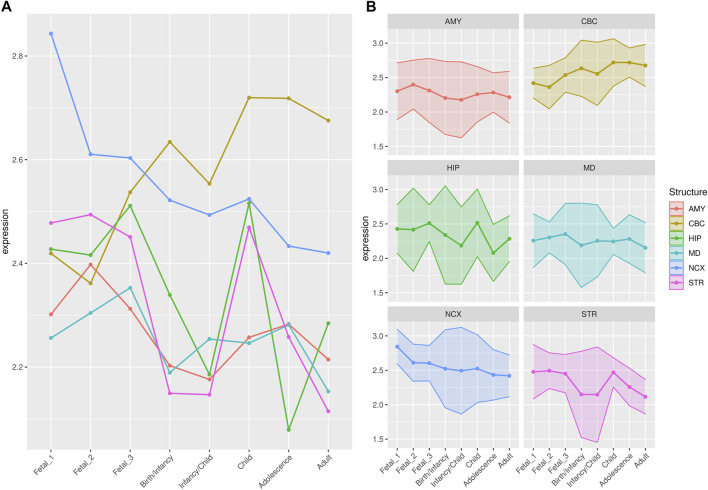
Median expression of genes under putative positive selection within large deserts. **(A)** Comparison of median of gene expression across developmental stages between structures and **(B)** Standard deviation per structure, for genes (*n* = 12) within the four large deserts of introgression and under putative positive selection. Genes expressed in neocortical areas reach the highest expression at the early fetal stages, whereas the cerebellar cortex, from birth until adulthood, retains its status as the structure with the highest expression profile. The expression profile of each of the twelve genes under putative positive selection within deserts is shown in Supplementary Figure S13. *Structures:* AMY, amygdala; CBC, cerebellar cortex; HIP, hippocampus; MD, mediodorsal nucleus of thalamus; NCX, neocortex; STR, striatum. *Stages:* Fetal 1: 12-13 post conception weeks (PCWs); Fetal 2: 16-18 PCW; Fetal 3: 19-22 PCW; Birth—Infancy: 35-37 PCW and 0–0.3 years; Infancy—Child: 0.5–2.5 years; Childhood: 2.8–10.7 years, Adolescence: 13–19 years; Adulthood: 21–64 years [as in (Li et al., 2018)].

In order to statistically evaluate the differences observed for each structure and developmental stage (see Figures 2, 3), we performed a Principal Component Analysis and calculated the pairwise Euclidean distances between brain regions for each developmental stage using statistically significant principal components (*p* < 0.05) as assessed using the JackStraw analysis implemented in Seurat (Butler et al., 2018). For genes within large deserts of introgression overlapping putative positively-selected regions, we performed dimensionality reduction on the first two principal components. Due to the low number of genes at this intersection (*n* = 12), the second principal component did not report statistical significance. The sum of the percentage of variance explained by first and second components is around 50%. The transcriptomic profile of a brain region in a given developmental stage was considered “divergent” if the expression value of the subset of genes under consideration was significantly different (*p* < 0.01) in that region when compared to all other regions (performing a Wilcoxon rank sum test with Bonferroni correction).

**FIGURE 4:**
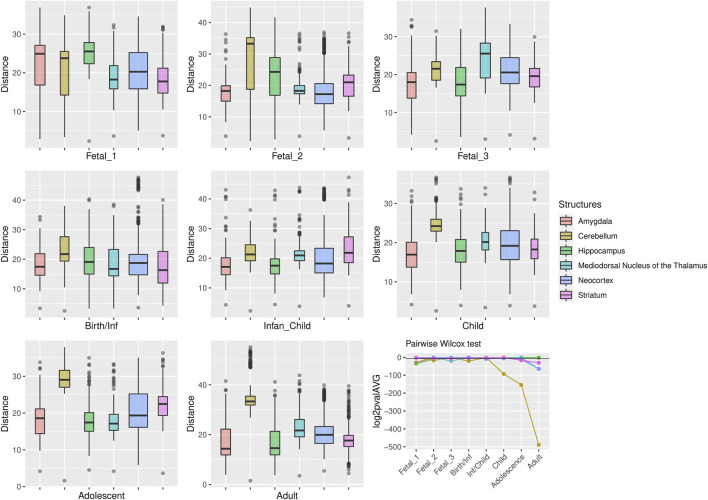
Genes within large deserts. One structure, the cerebellum, reports the most significant differences (pairwise Wilcoxon test with Bonferroni correction) encompassing postnatal stages: childhood (*p* = 1.06 × 10^−28^), adolescence (*p* = 4.71 × 10^−47^), adulthood (*p* = 5.54 × 10^−148^); also at birth (*p* = 5.64 × 10^−7^) and fetal stage 2 (*p* = 4, 79 × 10^−6^). Significant differences are also found for the thalamus (fetal stage 3 and adulthood), the hippocampus (fetal stages 1 and 2) or the striatum (fetal stage 3, adolescence and adulthood). The boxplots show the values of the pairwise Euclidean distances at each stage for each structure. The line graph represents the average *p*-value (log2-transformed for representational purposes) for each pairwise comparison between structures at each stage. The horizontal black line denotes *p* = 0.01.

**FIGURE 5:**
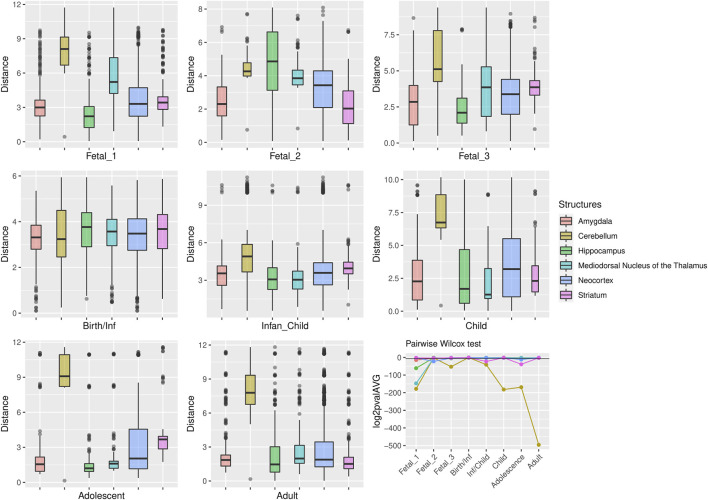
Genes under positive selection within large deserts. The two first principal components were selected to calculate the pairwise comparisons between structures at each stage (Wilcoxon test with Bonferroni correction). Significant results were obtained for the cerebellum more prominently at childhood *p* = 1.64 × 10^−55^, adolescence *p* = 1.45 × 10^−51^, and adulthood *p* = 5.83 × 10^−150^. Prenatally the cerebellum and the thalamus show the most significant differences in comparison to the rest of structures, at fetal stage 1 (*p* = 2.1 × 10^54^ and *p* = 4.47 × 10^−45^, respectively). Other significant results are found at specific stages for the striatum (infancy and adolescence), neocortex (fetal stage 2), or the amygdala (fetal stages 1 and 3). The horizontal black line in the line plot denotes *p* = 0.01.

For genes that reside in the deserts of introgression under consideration, the cerebellum stands out as the structure with the most divergent transcriptomic profile at postnatal stages, from childhood to adulthood (Figure 4). For genes under positive selection that are also found within introgression deserts, the cerebellum still remains as the most transcriptomically divergent structure postnatally (birth/infancy, childhood, adolescence and adulthood; see the caption of Figure 2 for the specific time points associated to each developmental stage). Moreover, prenatally, the cerebellum again (fetal stages 1 and 2) and the mediodorsal nucleus of the thalamus (fetal stage 1; see Supplementary Figures S2, S3) exhibit the most significant differences in the pairwise comparisons. Previous research found that genes within large deserts are over-represented in the striatum at adolescence and adult stages (Vernot et al., 2016). In agreement with this finding, we found that the transcriptomic profile of the striatum for genes within large deserts is significantly different at adolescence and adulthood but also at fetal stage 3, while for genes within deserts under putative positive selection, significant differences are found at infancy and adolescence (see Figures 4, 5, and Supplementary Figure S4). Lastly, to disentangle the effect of set of genes within specific chromosomes, we also evaluated the expression dynamics of genes within large deserts of introgression for each of the four chromosomal regions separately (a corresponding evaluation of the twelve genes under putative postive selection within deserts is presented in the next section). Overall, and in agreement with the previous observations, the cerebellum (at perinatal and later postnatal stages for the four chromosomes) and the striatum (at adulthoood for three out of four chromosomes, and childhood for one chromosomal region) are found as the most transcriptomically divergent structures. The transcriptomic profile of the mediodorsal nucleus of the thalamus was also found to be statistically different at fetal stages for chromosome 1 and chromosome 8 (see Supplementary Figures S5–S8).

For the sake of comparison, we note that a similar profile postnatally was obtained for the cerebellum when subsetting for genes under positive selection not present within large introgression deserts (marked differences from childhood to adulthood; see Supplementary Figure S9). When evaluating the global expression profile (*n* = 9,358 genes), the cerebellum shows statistically significant differences also at postanatal stages (birth, infancy, childhood and adulthood) and the mediodorsal nucleus of the thalamus at fetal stage 3 and adulthood (see Supplementary Figure S10). All *p*-values can be found in the Supplementary files.

The trajectories of expression across developmental stages in genes within large deserts of introgression might be affected by positive selection. To control for this, we analyzed the contrast between a control group of genes not under positive selection but within deserts of introgression compared to those under positive selection in these same regions. We found that, within large deserts of introgression, genes under positive selection have an overall lower expression than those in regions not under positive selection (*p* = 0.0007, Kruskal-Wallis test). A linear regression model predicts that this effect is not structure-specific (*p* = 0.655), and that overall variability in the data is not explained by between-structure differences (*p* = 0.9904, anova test between fitted models that do and do not include brain regions as a variable). Expression linked to specific developmental stages diverges significantly between genes under positive selection and those that are not (0.0001, linear regression). However, a post-hoc Tukey test (corrected for repeated measures, Supplementary Figure S11) reveals that this difference holds only at the fetal stages. In portions of large deserts not under selection, the fetal period of development is significantly different from most posterior stages, while in genes under selective pressures only the first fetal stage is significantly different from post-fetal stages (with a significance threshold of *p* < 0.05).

### 2.3 Gene-specific Expression Trajectories of Genes in the Overlapping Desertic and Positively-Selected Regions

As described in section 4, we included in our analyses any outlier present in the set of genes that are either within the four large deserts of introgression or under putative positive selection within large deserts, due to their potential evolutionary relevance. To evaluate in more detail the expression of specific genes, we focused on the specific trajectories of genes at the intersection of large deserts and positively-selected regions (*n* = 12 genes; Supplementary Figure S13), and performed a segmented regression analysis (using the Trendy package (Bacher et al., 2018)) filtering out genes with an adjusted *R* ^2^ less than 0.5. As our analysis showed a marked increase of transcriptomic divergence at different developmental stages for the cerebellum, the striatum and the mediodorsal nucleus of the thalamus, we decided to focus on these structures.

**FIGURE 6:**
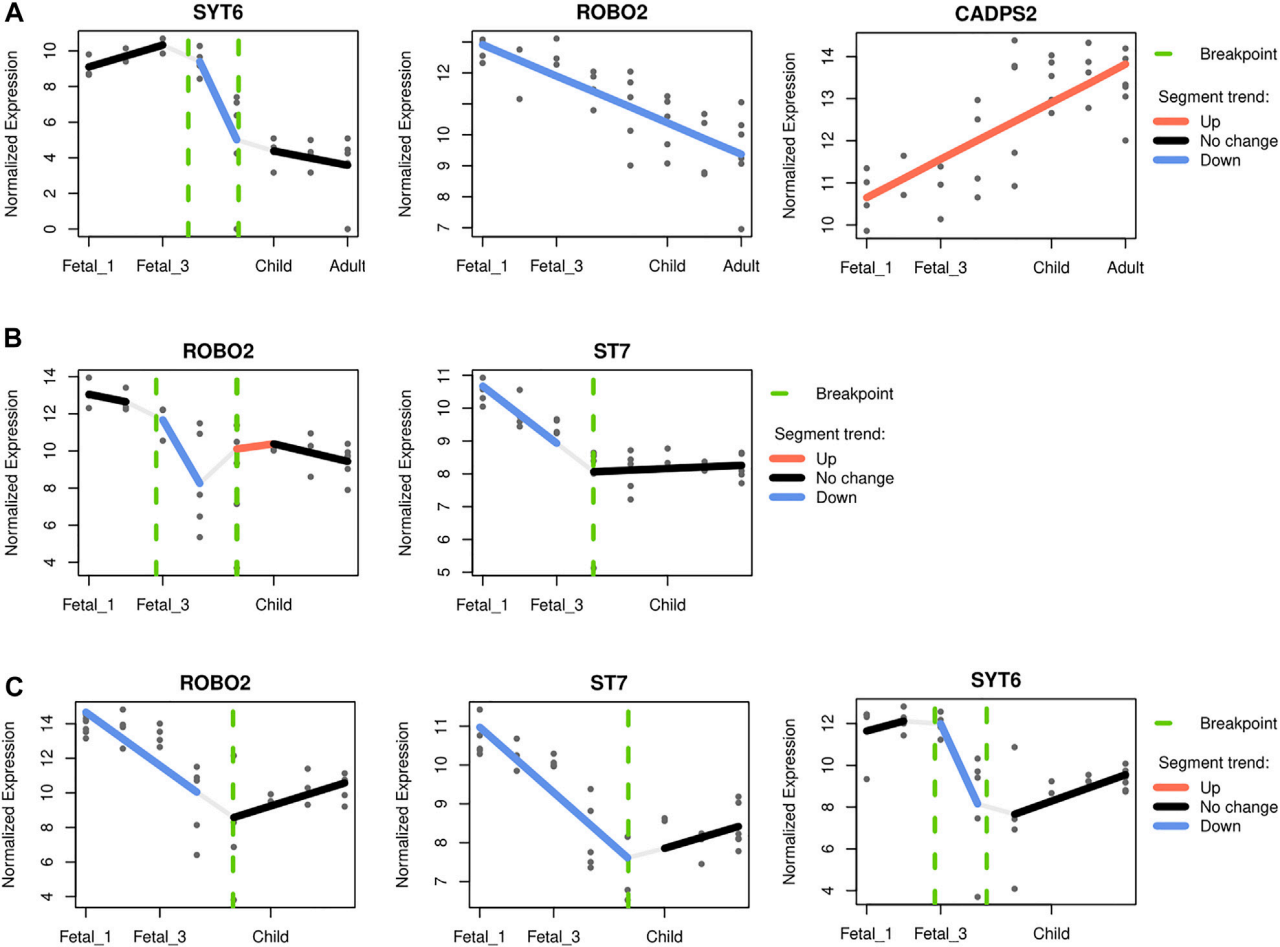
Gene-specific trajectories. A segmented regression analysis (Bacher et al., 2018) was performed to characterize the expression dynamics of genes under putative positive selection within introgression deserts, across developmental stages. Only genes with an adjusted *R* ^2^ above 0.5 were considered. **(A)** The cerebellum and the profile of *SYT6*, *ROBO2* and *CADPS2*. The profile of *CADPS2* was found to closely recapitulate the pattern observed for the cerellebum increasing from prenatal to postnatal stages (see Figure 3). **(B)** The mediodorsal nucleus of thalamus, and *ROBO2* and *ST7*, both genes with higher prenatal expression declining at around birth. **(C)** The striatum and *ROBO2*, *ST7* and *SYT6*. The three genes follow a similar expression dynamics peaking at early fetal stages but declining afterwards, and increasing again postnatally.

For the cerebellum, *CADPS2* (chromosome 7) expression is the one that most closely mimics the observed pattern, with highest postnatal expression and a marked increased of its expression around birth and infancy (*R* ^2^ 0.56; see Supplementary Figure S13 and Figure 6A). This Ca^2+^-dependent activator protein is known to regulate exocytosis in granule cells, particularly neurotrophic factors BDNF and NT-3 release, and its knockout disrupts normal cerebellar development and causes an autistic-like behavioral phenotype in mice (Sadakata et al., 2007; Sadakata et al., 2014). In addition, decreasing expression through developmental stages was also found for *SYT6* and *ROBO2* (chromosome 1 and 3 respectively; *R* ^2^ 0.76 and 0.60; see Figure 6A). Two other genes, *KCND2* and *ST7* (both in chromosome 7), exhibited comparatively high expression postnatally, but did not pass the adjusted *R* ^2^ threshold (Supplementary Figure S13). Regarding the thalamus, two genes within the overlapping desertic and positively-selected regions could be fitted with an adjusted *R* ^2^ higher than 0.5: *ROBO2* and *ST7*. Both genes show higher expressions at prenatal stages, followed by a steady decline at around birth (*R* ^2^ 0.65 and 0.61, respectively; see Figure 6B). The roles of Robo2 in the thalamus have been studied as a receptor of the Slit/Robo signaling pathway which is critically involved in axon guidance. Indeed, Robo2 is highly expressed in the dorsal thalamus and cerebral cortex in the embryonic mouse brain and, in cooperation with Robo1, is required for the proper development of cortical and thalamic axonal projections (López-Bendito et al., 2007).

Lastly, for the striatum, three genes within the overlapping desertic and positively-selected regions could be fitted with an adjusted *R* ^2^ higher than 0.5. *ST7*, *ROBO2* and *SYT6* follow a V-shape profile with higher expression at prenatal stages, a decrease around birth, and increasing levels during later postnatal stages (*R* ^2^ = 0.75, 0.57, and 0.53, respectively; see Figure 6C). While the role of *ST7* in neurodevelopment remains to be elucidated, Robo2 is a receptor of the Slit/Robo signaling pathway which is critically involved in axon guidance (López-Bendito et al., 2007), but also in the proliferation and differentiation of neural progenitors with possible different roles in dorsal and ventral telencephalon (Andrews et al., 2008; Borrell et al., 2012). *Syt6* is another synapse-related gene expressed in the developing basal ganglia (Long et al., 2009), and in fact linked to the distinctive expression profile of this structure (Konopka et al., 2012). Additionally, *Syt6* shows a similar expression profile in the cerebellar cortex although at lower levels (see Figure 6C), a region where *Syt6* has been found, in mice, to be differentially expressed in a *Cadps2* knockout background (Sadakata et al., 2017).

## 3 Discussion

There are two main findings to take away from our study: the importance of structures beyond the cerebral neocortex in the attempt to characterize some of the most derived features of our species’ brain, and the fact that some of the strongest effects in these regions takes place at early stages of development. In this way our work provides complementary evidence for the perinatal globularization phase as a species-specific ontogenic innovation (Gunz et al., 2010), and also provides new evidence for the claim that brain regions outside the neocortex (cerebellum, thalamus, striatum) significantly contribute to this phenotype (Boeckx and Benítez-Burraco, 2014; McCoy et al., 2017; Neubauer et al., 2018; Gunz et al., 2019; Weiss et al., 2021).

To our knowledge this is the first study to reveal the effect of the cerebellum in the context of large introgression deserts. For the striatum, previous studies have already highlighted the relevance of this structure: genes carrying Neanderthal-derived changes and expressed in the striatum during adolescence exhibit a higher McDonald-Kreitman ratio (Mafessoni et al., 2020). In addition, using a different range of introgressed regions and gene expression data from the Allen Brain Atlas (with lower temporal resolution than the database used in this study), it had already been noted (Vernot et al., 2016) that genes within large deserts are significantly enriched in the striatum at adolescence and adult stages, which converges with the life stages highlighted from our analysis using the most recent report of genomic regions depleted of archaic variants (Chen et al., 2020).

Naturally, the functional effects of these divergent developmental profiles for the cerebellum, the prenatal thalamus or the striatum remain to be understood, particularly in the context of the possible differences among *Homo*-species concerning regulation of the genes highlighted in this study. This is especially relevant in light of emerging evidence that selection against DNA introgression is stronger in regulatory regions (Vilgalys et al., 2021), which in addition have been found to be over-represented in putative positively-selected regions in *Homo sapiens* (Peyrégne et al., 2017; Petr et al., 2019). The fact that early developmental stages are critical holds the promise of using brain organoid technology to probe the nature of these differences, since such *in vitro* techniques best track these earliest developmental windows (Muchnik et al., 2019; Mostajo-Radji et al., 2020; Kyrousi and Cappello, 2020). Our level of analysis (mRNA-seq data, informed by paleogenetic studies) can be complemented with other *omics* data to finely resolve cell-type specificities of the genes considered here across brain areas, as with the use of single-cell RNA-seq data, or to infer gene regulatory networks (from differentially accessible and methylated regions and chromatin immunoprecipitation data) that underlie the divergent gene expression trajectories observed.

The fact that FOXP2 expression is known to be particularly high in the brain regions highlighted here (Lai et al., 2003) may help shed light on why *FOXP2* is found in one of the large introgression deserts in modern human genomes. As pointed out in (Kuhlwilm, 2018), this portion of chromosome 7 is not a desert for introgression in other great apes, nor did it act as a barrier for gene flow from Sapiens into Neanderthals. As such, it may indeed capture something genuinely specific about our species.

## 4 Methods

Analyses were performed using R (R Core Team, 2019). Putative positively-selected regions were retrieved from the extended set of sweep regions in Peyrégne et al. (2017), built from two independent recombination maps using a Hidden Markov-based model applied to African and Neaderthal/Denisovan genomes. Coordinates for (large) deserts of introgression were retrieved from Chen et al. (2020), and genes within these two sets of regions were obtained using the BioMart R package version 2.42.1 (Durinck et al., 2009), using the respective genomic region coordinates as input and filtering by protein-coding genes.

**mRNA-seq analysis**. Publicly available transcriptomic data of the human brain at different developmental stages was retrieved from (Li et al., 2018) and analyzed using R (full code can be found at https://github.com/jjaa-mp/desertsHomo). Reads per kilo base per million mapped reads (RPKM) normalized counts were log-transformed and then subsetted to select genes either in large deserts of introgression or in both deserts and putative positively-selected regions. The complete log-transformed, RPKM normalized count matrix was subsetted to select genes with median expression value >2 , as in (Li et al., 2018), while no median filtering was employed for the subsets of genes within deserts and positively-selected regions, due to the potential relevance of the outliers in these specific regions for the purposes of our study. To assess transcriptomic variability between brain regions accounted for by genes either in large deserts or in deserts and positively-selected regions, we performed principal component analysis and calculated the pairwise Euclidean distances between brain regions for each dataset [following (Li et al., 2018)]. We then statistically evaluated such differences at each developmental stage using pairwise Wilcoxon tests with Bonferroni correction. Significant differences were considered if *p* < 0.01. Our analysis based on statistically significant principal components did not make it possible for us to use the Allen Brain Atlas data for comparisons with the psychENCODE project dataset used in this study, due to the more limited resolution, especially at prenatal stages, offered by the former.

To evaluate the expression profile of genes from our regions of interest in comparison to other regions of the human genome, we generated sets of random regions of the same length and gene density (that do not overlap with the genomic coordinates of deserts on introgression). These served as control regions for comparisons of mean expression values using two-way repeated measures ANOVA, implemented in R. ANOVA tests were performed taking mean expression values as dependent value, with structure names as subject identifiers and the different regions of interest (datasource) as between-subjects factor variable. Posthocs tests were performed similarly but with the mean expression data grouped by the datasource, obtaining an ANOVA table for each structure, with a Bonferroni correction to account for repeated measures. The stage-version of the ANOVA grouped subject identifiers by stage. Two Kruskal-Wallis tests were used, one designed to detect whether non positively-selected genes in deserts of introgression have different mean expression levels than genes that are both in deserts and in positively-selected windows; and the second to determine whether any particular brain structure has a particularly different expression mean than the rest, regardless of selection. We also used two two-level linear mixed-effects regression models, to compare non-positively selected genes and positively selected genes within introgression deserts. These models consist of repeated measures of expression on different brain structures in three different groups: control, deserts of introgression, and deserts with selection signals. The same model applies when stages are taken into account, replacing structure identifiers. Tukey’s test was then used to fit the model.

**Gene-specific expression trajectories**. The R package Trendy version 1.8.2 (Bacher et al., 2018) was used to perform segmented regression analysis and characterize the expression trajectories of genes within both deserts of introgression and putative positively-selected regions (12 genes). The normalized RPKM values [from (Li et al., 2018)] in the form of a gene-by-time samples matrix was used to fit each gene expression trajectory to an optimal segmented regression model. Genes were considered if their adjusted *R* ^2^ was >0.5 . In addition, a maximum number of breakpoints (significant changes in gene expression trajectory) was set at 3, minimum number of samples in each segment at 2, and minimum mean expression, 2.

The permutation tests using gene expression data from (Li et al., 2018) were done using the regioneR package version 1.26.1 (Gel et al., 2016) at *n* = 1,000.

## Data Availability

Publicly available datasets were analyzed in this study. This data can be found here: https://github.com/jjaa-mp/desertsHomo.

## References

[B1] Andrews W. Barber M. Hernadez-Miranda L. R. Xian J. Rakic S. Sundaresan V. (2008). The Role of Slit-Robo Signaling in the Generation, Migration and Morphological Differentiation of Cortical Interneurons. Develop. Biol. 313, 648–658. 10.1016/j.ydbio.2007.10.052 18054781

[B2] Bacher R. Leng N. Chu L.-F. Ni Z. Thomson J. A. Kendziorski C. (2018). Trendy: Segmented Regression Analysis of Expression Dynamics in High-Throughput Ordered Profiling Experiments. BMC Bioinformatics 19, 380. 10.1186/s12859-018-2405-x 30326833 PMC6192113

[B3] Bergström A. Stringer C. Hajdinjak M. Scerri E. M. L. Skoglund P. (2021). Origins of Modern Human Ancestry. Nature 590, 229–237. 10.1038/s41586-021-03244-5 33568824

[B4] Boeckx C. A. Benítez-Burraco A. (2014). The Shape of the Human Language-Ready Brain. Front. Psychol. 5. 10.3389/fpsyg.2014.00282 PMC3983487 24772099

[B5] Borrell V. Cárdenas A. Ciceri G. Galcerán J. Flames N. Pla R. (2012). Slit/Robo Signaling Modulates the Proliferation of Central Nervous System Progenitors. Neuron 76, 338–352. 10.1016/j.neuron.2012.08.003 23083737 PMC4443924

[B6] Butler A. Hoffman P. Smibert P. Papalexi E. Satija R. (2018). Integrating Single-Cell Transcriptomic Data across Different Conditions, Technologies, and Species. Nat. Biotechnol. 36, 411–420. 10.1038/nbt.4096 29608179 PMC6700744

[B7] Chen L. Wolf A. B. Fu W. Li L. Akey J. M. (2020). Identifying and Interpreting Apparent Neanderthal Ancestry in African Individuals. Cell 180, 677–687. e16. 10.1016/j.cell.2020.01.012 32004458 PMC12805117

[B8] Durinck S. Spellman P. T. Birney E. Huber W. (2009). Mapping Identifiers for the Integration of Genomic Datasets with the R/Bioconductor Package biomaRt. Nat. Protoc. 4, 1184–1191. 10.1038/nprot.2009.97 19617889 PMC3159387

[B9] Eising E. Mirza-Schreiber N. de Zeeuw E. L. Wang C. A. Truong D. T. Allegrini A. G. (2021). Genome-wide Association Analyses of Individual Differences in Quantitatively Assessed reading- and Language-Related Skills in up to 34,000 People. *bioRxiv* 10.1101/2021.11.04.466897 PMC9436320 35998220

[B10] Fisher S. E. (2019). Human Genetics: The Evolving Story of FOXP2. Curr. Biol. 29, R65–R67. 10.1016/j.cub.2018.11.047 30668952

[B11] Fontsere C. Manuel M. d. Marques-Bonet T. Kuhlwilm M. (2019). Admixture in Mammals and How to Understand its Functional Implications. BioEssays 41, 1900123. 10.1002/bies.201900123 31664727

[B12] Gel B. Díez-Villanueva A. Serra E. Buschbeck M. Peinado M. A. Malinverni R. (2016). regioneR: an R/Bioconductor Package for the Association Analysis of Genomic Regions Based on Permutation Tests. Bioinformatics 32, 289–291. 10.1093/bioinformatics/btv562 26424858 PMC4708104

[B13] Gunz P. Neubauer S. Maureille B. Hublin J.-J. (2010). Brain Development after Birth Differs between Neanderthals and Modern Humans. Curr. Biol. 20, R921–R922. 10.1016/j.cub.2010.10.018 21056830

[B14] Gunz P. Tilot A. K. Wittfeld K. Teumer A. Shapland C. Y. van Erp T. G. (2019). Neandertal Introgression Sheds Light on Modern Human Endocranial Globularity. Curr. Biol. 29, 120–127. e5. 10.1016/j.cub.2018.10.065 30554901 PMC6380688

[B15] Hajdinjak M. Mafessoni F. Skov L. Vernot B. Hübner A. Fu Q. (2021). Initial Upper Palaeolithic Humans in Europe Had Recent Neanderthal Ancestry. Nature 592, 253–257. 10.1038/s41586-021-03335-3 33828320 PMC8026394

[B16] Iasi L. N. M. Ringbauer H. Peter B. M. (2021). An Extended Admixture Pulse Model Reveals the Limitations to Human–Neandertal Introgression Dating. Mol. Biol. Evol. 10.1093/molbev/msab210 PMC8557420 34254144

[B17] Konopka G. Friedrich T. Davis-Turak J. Winden K. Oldham M. C. Gao F. (2012). Human-Specific Transcriptional Networks in the Brain. Neuron 75, 601–617. 10.1016/j.neuron.2012.05.034 22920253 PMC3645834

[B18] Kuhlwilm M. Han S. Sousa V. C. Excoffier L. Marques-Bonet T. (2019). Ancient Admixture from an Extinct Ape Lineage into Bonobos. Nat. Ecol. Evol. 3, 957–965. 10.1038/s41559-019-0881-7 31036897

[B19] Kuhlwilm M. (2018). The Evolution of FOXP2 in the Light of Admixture. Curr. Opin. Behav. Sci. 21, 120–126. 10.1016/j.cobeha.2018.04.006

[B20] Kyrousi C. Cappello S. (2020). Using Brain Organoids to Study Human Neurodevelopment, Evolution and Disease. WIREs Develop. Biol. 9, e347. 10.1002/wdev.347 31071759

[B21] Lai C. S. L. Fisher S. E. Hurst J. A. Vargha-Khadem F. Monaco A. P. (2001). A Forkhead-Domain Gene Is Mutated in a Severe Speech and Language Disorder. Nature 413, 519–523. 10.1038/35097076 11586359

[B22] Lai C. S. L. Gerrelli D. Monaco A. P. Fisher S. E. Copp A. J. (2003). FOXP2 Expression during Brain Development Coincides with Adult Sites of Pathology in a Severe Speech and Language Disorder. Brain 126, 2455–2462. 10.1093/brain/awg247 12876151

[B23] Li M. Santpere G. Kawasawa Y. I. Evgrafov O. V. Gulden F. O. Pochareddy S. (2018). Integrative Functional Genomic Analysis of Human Brain Development and Neuropsychiatric Risks. Science 362. 10.1126/science.aat7615 PMC6413317 30545854

[B24] Long J. E. Cobos I. Potter G. B. Rubenstein J. L. R. (2009). Dlx1&2 and Mash1 Transcription Factors Control MGE and CGE Patterning and Differentiation through Parallel and Overlapping Pathways. Cereb. Cortex 19, i96–i106. 10.1093/cercor/bhp045 19386638 PMC2693539

[B25] López-Bendito G. Flames N. Ma L. Fouquet C. Meglio T. D. Chedotal A. (2007). Robo1 and Robo2 Cooperate to Control the Guidance of Major Axonal Tracts in the Mammalian Forebrain. J. Neurosci. 27, 3395–3407. 10.1523/JNEUROSCI.4605-06.2007 17392456 PMC6672128

[B26] Mafessoni F. Grote S. Filippo C. d. Slon V. Kolobova K. A. Viola B. (2020). A High-Coverage Neandertal Genome from Chagyrskaya Cave. Proc. Natl. Acad. Sci. 117, 15132–15136. 10.1073/pnas.2004944117 32546518 PMC7334501

[B27] Martin S. H. Jiggins C. D. (2017). Interpreting the Genomic Landscape of Introgression. Curr. Opin. Genet. Develop. 47, 69–74. 10.1016/j.gde.2017.08.007 28923541

[B28] McCoy R. C. Wakefield J. Akey J. M. (2017). Impacts of Neanderthal-Introgressed Sequences on the Landscape of Human Gene Expression. Cell 168, 916–927. e12. 10.1016/j.cell.2017.01.038 28235201 PMC6219754

[B29] Mekki Y. Guillemot V. Lemaître H. Carrión-Castillo A. Forkel S. Frouin V. (2022). The Genetic Architecture of Language Functional Connectivity. NeuroImage 249, 118795. 10.1016/j.neuroimage.2021.118795 34929384

[B30] Meyer M. Kircher M. Gansauge M.-T. Li H. Racimo F. Mallick S. (2012). A High-Coverage Genome Sequence from an Archaic Denisovan Individual. Science 338, 222–226. 10.1126/science.1224344 22936568 PMC3617501

[B31] Mostajo-Radji M. A. Schmitz M. T. Montoya S. T. Pollen A. A. (2020). Reverse Engineering Human Brain Evolution Using Organoid Models. Brain Res. 1729, 146582. 10.1016/j.brainres.2019.146582 31809699 PMC7058376

[B32] Muchnik S. K. Lorente-Galdos B. Santpere G. Sestan N. (2019). Modeling the Evolution of Human Brain Development Using Organoids. Cell 179, 1250–1253. 10.1016/j.cell.2019.10.041 31778651 PMC7034679

[B33] Neubauer S. Hublin J.-J. Gunz P. (2018). The Evolution of Modern Human Brain Shape. Sci. Adv. 4, eaao5961. 10.1126/sciadv.aao5961 29376123 PMC5783678

[B34] Pääbo S. (2014). The Human Condition—A Molecular Approach. Cell 157, 216–226. 10.1016/j.cell.2013.12.036 24679537

[B35] Petr M. Pääbo S. Kelso J. Vernot B. (2019). Limits of Long-Term Selection against Neandertal Introgression. Proc. Natl. Acad. Sci. 116, 1639–1644. 10.1073/pnas.1814338116 30647110 PMC6358679

[B36] Peyrégne S. Boyle M. J. Dannemann M. Prüfer K. (2017). Detecting Ancient Positive Selection in Humans Using Extended Lineage Sorting. Genome Res. 27, 1563–1572. 10.1101/gr.219493.116 28720580 PMC5580715

[B37] Prüfer K. Filippo C. d. Grote S. Mafessoni F. Korlević P. Hajdinjak M. (2017). A High-Coverage Neandertal Genome from Vindija Cave in Croatia. Science 358, 655–658. 10.1126/science.aao1887 28982794 PMC6185897

[B38] Prüfer K. Racimo F. Patterson N. Jay F. Sankararaman S. Sawyer S. (2014). The Complete Genome Sequence of a Neanderthal from the Altai Mountains. Nature 505, 43–49. 10.1038/nature12886 24352235 PMC4031459

[B39] R Core Team (2019). R: A Language and Environment for Statistical Computing. Vienna, Austria: R Foundation for Statistical Computing.

[B40] Rinker D. C. Simonti C. N. McArthur E. Shaw D. Hodges E. Capra J. A. (2020). Neanderthal Introgression Reintroduced Functional Ancestral Alleles Lost in Eurasian Populations. Nat. Ecol. Evol. 4, 1332–1341. 10.1038/s41559-020-1261-z 32719451 PMC7529911

[B41] Sadakata T. Kakegawa W. Shinoda Y. Hosono M. Katoh-Semba R. Sekine Y. (2014). Axonal Localization of Ca2+-dependent Activator Protein for Secretion 2 Is Critical for Subcellular Locality of Brain-Derived Neurotrophic Factor and Neurotrophin-3 Release Affecting Proper Development of Postnatal Mouse Cerebellum. PLOS ONE 9, e99524. 10.1371/journal.pone.0099524 24923991 PMC4055771

[B42] Sadakata T. Shinoda Y. Ishizaki Y. Furuichi T. (2017). Analysis of Gene Expression in Ca2+-dependent Activator Protein for Secretion 2 (Cadps2) Knockout Cerebellum Using GeneChip and KEGG Pathways. Neurosci. Lett. 639, 88–93. 10.1016/j.neulet.2016.12.068 28041965

[B43] Sadakata T. Washida M. Iwayama Y. Shoji S. Sato Y. Ohkura T. (2007). Autistic-like Phenotypes in Cadps2-Knockout Mice and Aberrant CADPS2 Splicing in Autistic Patients. J. Clin. Invest. 117, 931–943. 10.1172/JCI29031 17380209 PMC1821065

[B44] Sankararaman S. Mallick S. Patterson N. Reich D. (2016). The Combined Landscape of Denisovan and Neanderthal Ancestry in Present-Day Humans. Curr. Biol. 26, 1241–1247. 10.1016/j.cub.2016.03.037 27032491 PMC4864120

[B45] Skov L. Coll Macià M. Sveinbjörnsson G. Mafessoni F. Lucotte E. A. Einarsdóttir M. S. (2020). The Nature of Neanderthal Introgression Revealed by 27,566 Icelandic Genomes. Nature 582, 78–83. 10.1038/s41586-020-2225-9 32494067

[B46] St Pourcain B. Cents R. A. M. Whitehouse A. J. O. Haworth C. M. A. Davis O. S. P. O’Reilly P. F. (2014). Common Variation Near ROBO2 Is Associated with Expressive Vocabulary in Infancy. Nat. Commun. 5, 4831. 10.1038/ncomms5831 25226531 PMC4175587

[B47] Veller C. Edelman N. B. Muralidhar P. Nowak M. A. (2021). Recombination and Selection against Introgressed DNA. bioRxiv 10.1101/846147 36775972

[B48] Vernot B. Tucci S. Kelso J. Schraiber J. G. Wolf A. B. Gittelman R. M. (2016). Excavating Neandertal and Denisovan DNA from the Genomes of Melanesian Individuals. Science 352, 235–239. 10.1126/science.aad9416 26989198 PMC6743480

[B49] Vilgalys T. P. Fogel A. S. Mututua R. S. Warutere J. K. Siodi L. Anderson J. A. (2021). Selection against Admixture and Gene Regulatory Divergence in a Long-Term Primate Field Study. bioRxiv. 10.1101/2021.08.19.456711 PMC9682493 35926022

[B50] Wang R. Chen C.-C. Hara E. Rivas M. V. Roulhac P. L. Howard J. T. (2015). Convergent Differential Regulation of SLIT-ROBO Axon Guidance Genes in the Brains of Vocal Learners. J. Comp. Neurol. 523, 892–906. 10.1002/cne.23719 25424606 PMC4329046

[B51] Wang S. Rohwer S. Zwaan D. R. d. Toews D. P. L. Lovette I. J. Mackenzie J. (2020). Selection on a Small Genomic Region Underpins Differentiation in Multiple Color Traits between Two Warbler Species. Evol. Lett. 4, 502–515. 10.1002/evl3.198 33312686 PMC7719548

[B52] Weiss C. V. Harshman L. Inoue F. Fraser H. B. Petrov D. A. Ahituv N. (2021). The Cis-Regulatory Effects of Modern Human-specific Variants. eLife 10, e63713. 10.7554/eLife.63713 33885362 PMC8062137

[B53] Wolf A. B. Akey J. M. (2018). Outstanding Questions in the Study of Archaic Hominin Admixture. PLOS Genet. 14, e1007349. 10.1371/journal.pgen.1007349 29852022 PMC5978786

