# Supplementary material for: A Brain Region-Specific Expression Profile for Genes Within Large Introgression Deserts and Under Positive Selection in Homo sapiens
Source: Front Cell Dev Biol. 2022 Apr 26;10:824740. doi: 10.3389/fcell.2022.824740 (PMC9086289; doi:10.3389/fcell.2022.824740)
Supplement: Supplementary file 1 [file DataSheet1.PDF]

Supplementary Material

for

“A brain region-specific expression profile for genes within large introgression deserts and under positive selection in *Homo sapiens*”

Raül Buisan[1,*], Juan Moriano[1,2,*], Alejandro Andirkó[1,2], and Cedric Boeckx[1,2,3,**]

[1]Universitat de Barcelona

[2]Universitat de Barcelona Institute of Complex Systems

[3]Catalan Institute for Research and Advanced Studies (ICREA)

[*]Contributed equally

[**]Correspondence: cedric.boeckx@ub.edu

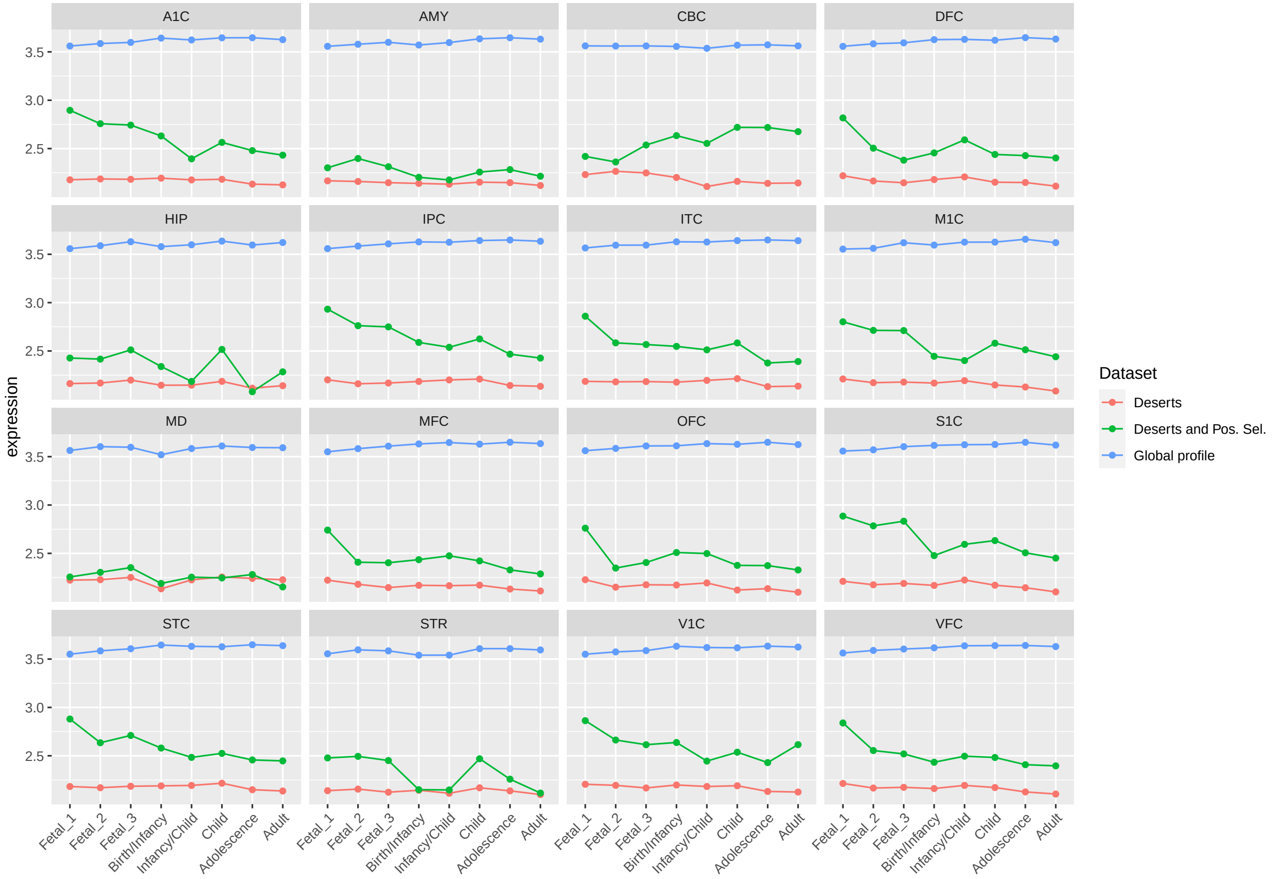

Figure 1: **Median expression profile of genes within large deserts, deserts/positively-selected regions and the global dataset, across structures and stages.** Genes within deserts/positively-selected regions have higher expression in comparison to the broader subset of genes within deserts, with a peak of expression at prenatal stages in neocortical areas. This peak is not observed in non-neocortical areas and, in the case of the cerebellar cortex, the genes (within deserts/positively-selected regions) show increasing expression at postnatal stages.

As explained in *Methods* section, the log-transformed expression values for the global profile was filtered by setting a threshold of median expression value > 2, as in the original publication [1], since the inclusion of too many zeros makes the determination of trajectories unreliable. However, for genes from our regions of interest, no threshold was set in order to detect outliers of potential relevance.

A1C, primary auditory cortex; AMY, amygdala; CBC, cerebellar cortex; DFC, dorsolateral prefrontal cortex; HIP, hippocampus; IPC, posterior inferior parietal cortex; ITC, inferior temporal cortex; M1C, primary motor cortex; MD, mediodorsal nucleus of thalamus; MFC, medial prefrontal cortex; OFC, orbital prefrontal cortex; S1C, primary somatosensory cortex; STC, superior temporal cortex; STR, striatum; V1C, primary visual cortex; VFC, ventrolateral prefrontal cortex.

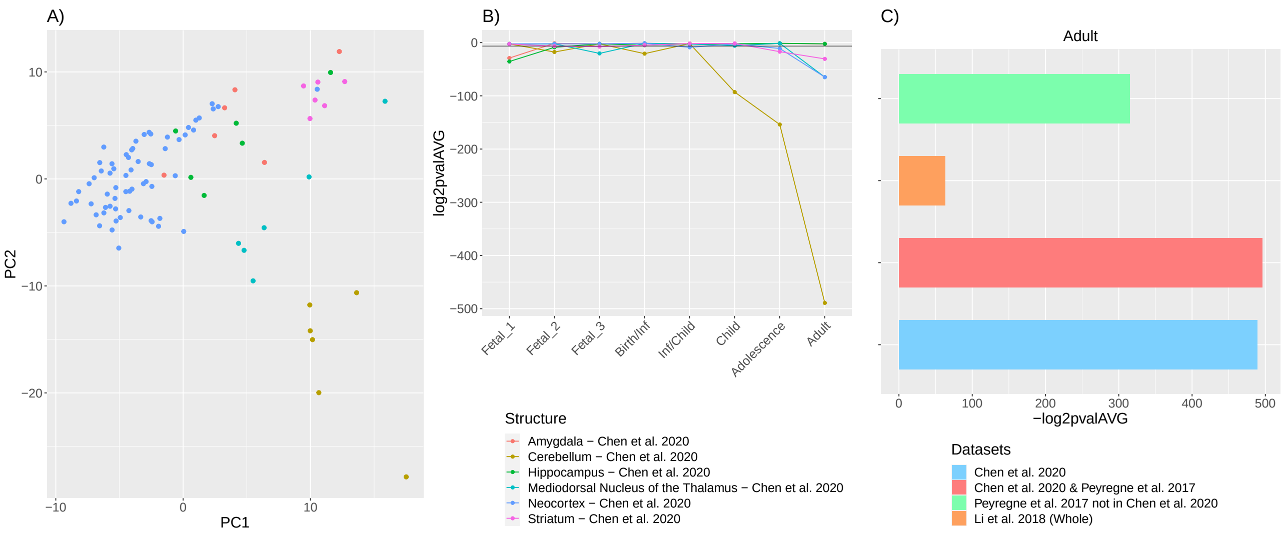

Figure 2: **The cerebellum's transcriptomic profile significantly diverges at postnatal stages.** For genes within large deserts of introgression, the cerebellum exhibits the most significant differences when evaluating pairwise distances between structures based on the statistically significant principal components. A) Distribution of structures at the adult stage using the first two principal components. B) P-values (log2-transformed) obtained from pairwise comparisons among structures at each developmental stage (Wilcoxon rank sum test with Bonferroni correction). C) Contribution of genes within large deserts of introgression [7], deserts of introgression under putative positive selection [7, 35], regions under putative selection [35] not within large deserts, and the raw dataset used in this study [1] to the observed divergence at adult stage for the cerebellum, with the greatest value for genes within large deserts.

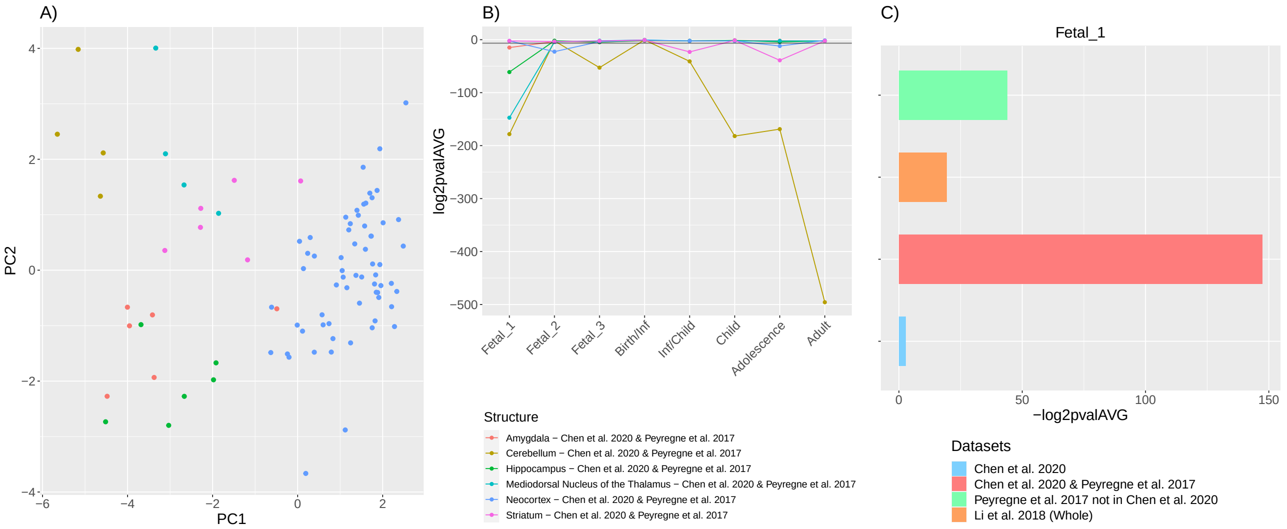

Figure 3: **The transcriptomic profile of the mediodorsal nucleus of the thalamus significantly diverges at fetal stage 1 for genes within deserts under positive selection.** A) Distribution of structures at the fetal stage 1 using the first two principal components. B) P-values (log2-transformed) obtained from pairwise comparisons among structures at each developmental stage (Wilcoxon rank sum test with Bonferroni correction). C) Contribution of genes within deserts of introgression [7], deserts of introgression under putative positive selection [7, 35], regions under putative selection [35] not within large deserts, and the raw dataset used in this study [1], to the observed divergence at adolescence for the striatum. The greatest value is found for genes within deserts under putative positive selection.

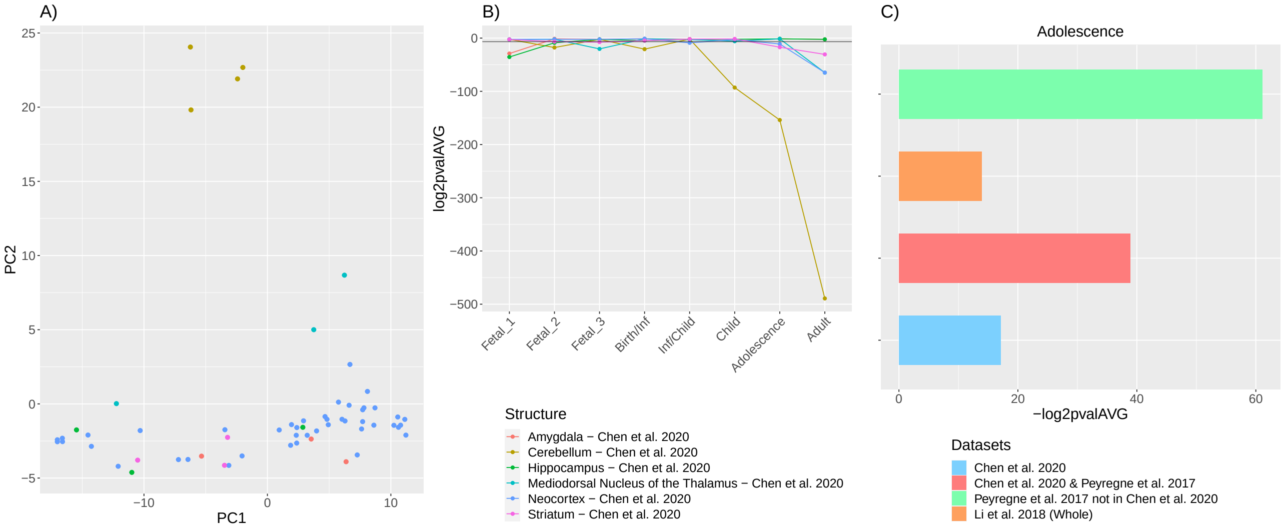

Figure 4: **The striatum's transcriptomic profile significantly diverges at adolescence for genes within large deserts.** When evaluating the transcriptome of genes within large deserts of introgression, the striatum reports significant differences postnatally at adolescence and adult stages. A) Distribution of structures at the adolescence using the first two principal components. B) P-values (log2-transformed) obtained from pairwise comparisons among structures at each developmental stage (Wilcoxon rank sum test with Bonferroni correction). C) Contribution of genes within large deserts of introgression [7], deserts of introgression under putative positive selection [7, 35], regions under putative selection [35] not within large deserts, and the raw dataset used in this study [1], to the observed divergence at adolescence for the striatum. The greatest value is found for genes under putative positive selection not within deserts of introgression, an effect also shown in Supplementary Figure 9.

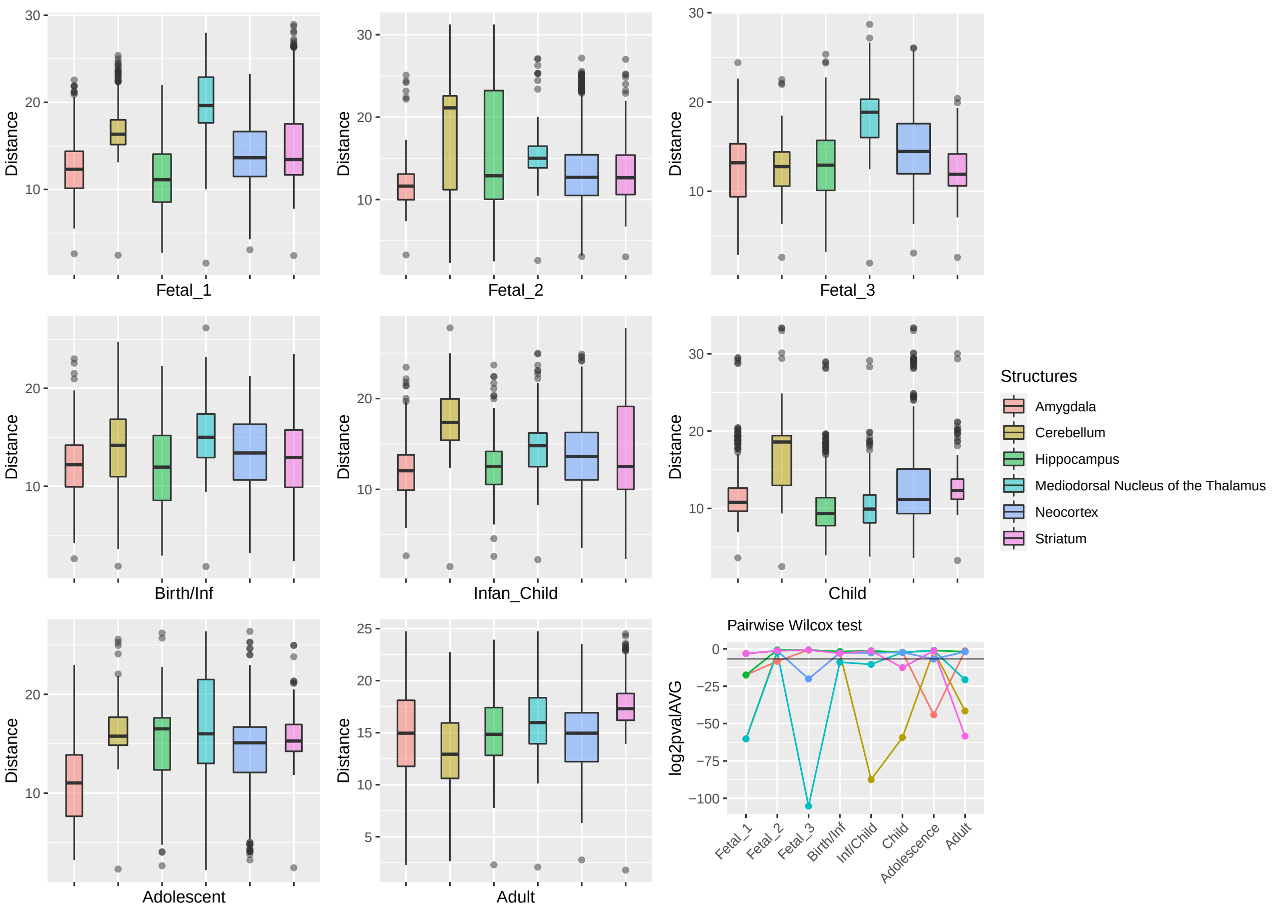

Figure 5: **Evaluation of transcriptomic divergence considering genes within deserts for chromosome 1.** These and the following Supplementary Figures 6, 7 and 8 show the expression of genes within large deserts for each of the chromosomes harboring deserts of introgression, as reported in [7]. For genes within chromosome 1 desert (n = 132), the sharpest difference is observed for the mediodorsal nucleus of the thalamus at fetal stage 3. Other structures that show marked statistically significant differences are the cerebellum (fetal stage 1, infancy/childhood, childhood, adulthood), the amygadala (adolescence) and the striatum (adulthood). All p-values can be found at the online repository `https://github.com/jjaa-mp/desertsHomo` .

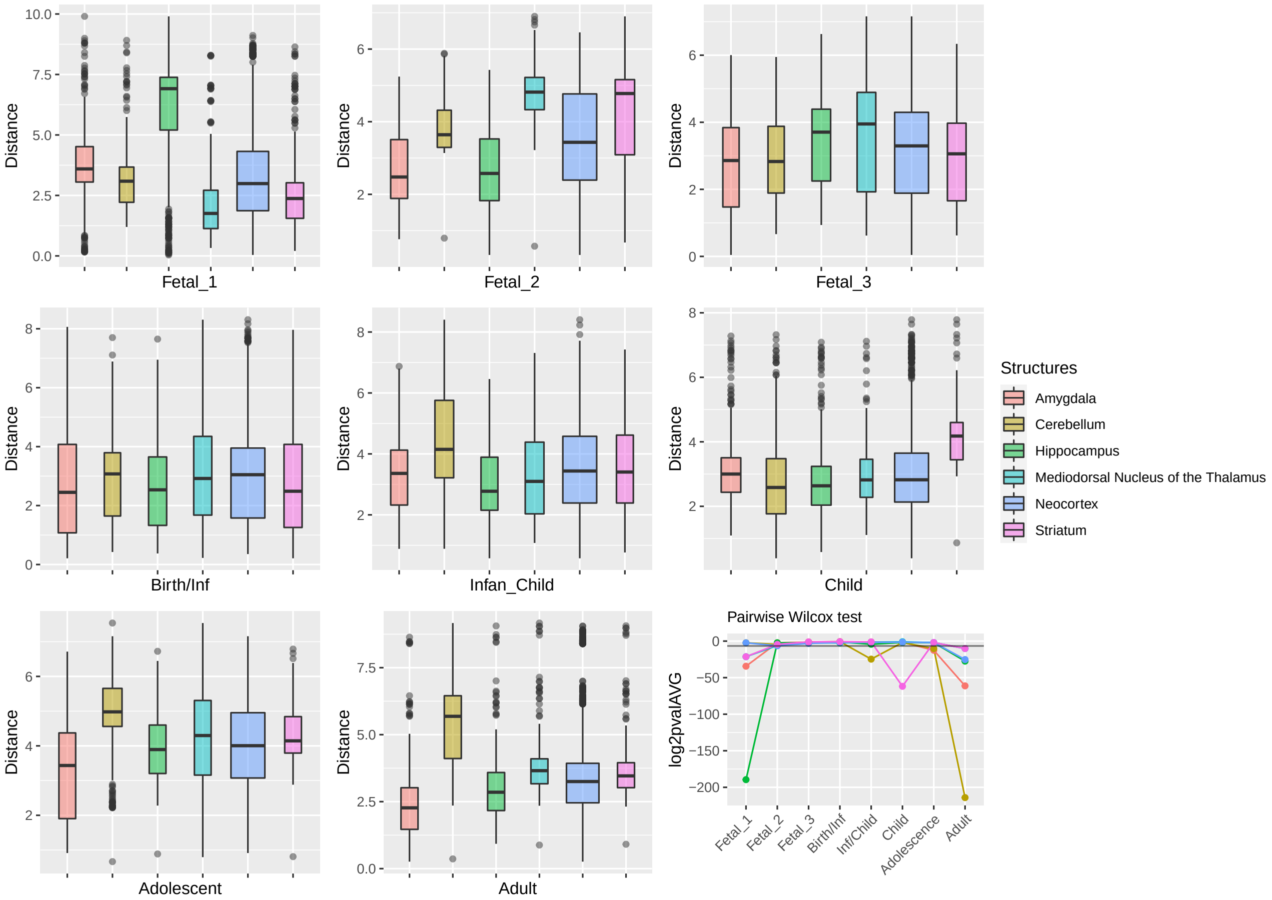

Figure 6: **Evaluation of transcriptomic divergence considering genes within deserts for chromosome 3.** For genes within chromosome 3 desert (n = 15), the hippocampus at fetal stage 1 and the cerebellum at adulthood are the most transcriptomically divergent structures, followed by the striatum (childhood) and the amygdala (adulthood). All p-values can be found at the online repository `https://github.com/jjaa-mp/desertsHomo`

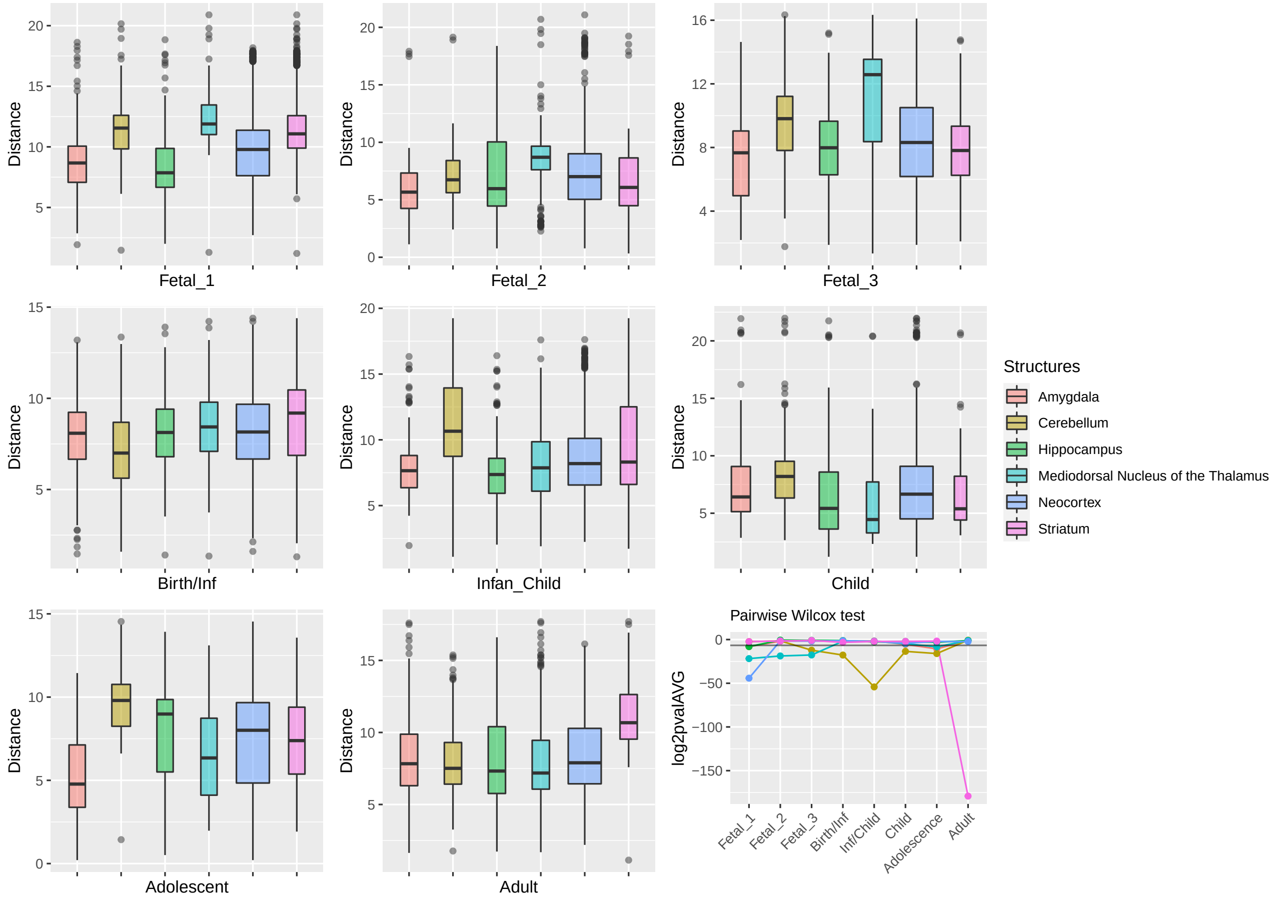

Figure 7: **Evaluation of transcriptomic divergence considering genes within deserts for chromosome 7.** In chromosome 7 desert (n = 62), the striatum is found as the most significantly different transcriptome at adulthood. The mediodorsal nucleus of the thalamus (fetal stages) and the cerebellum (from fetal stage 3 and birth to adolescence) are also found as statistically divergent structures. All p-values can be found at the online repository `https://github.com/jjaa-mp/desertsHomo`

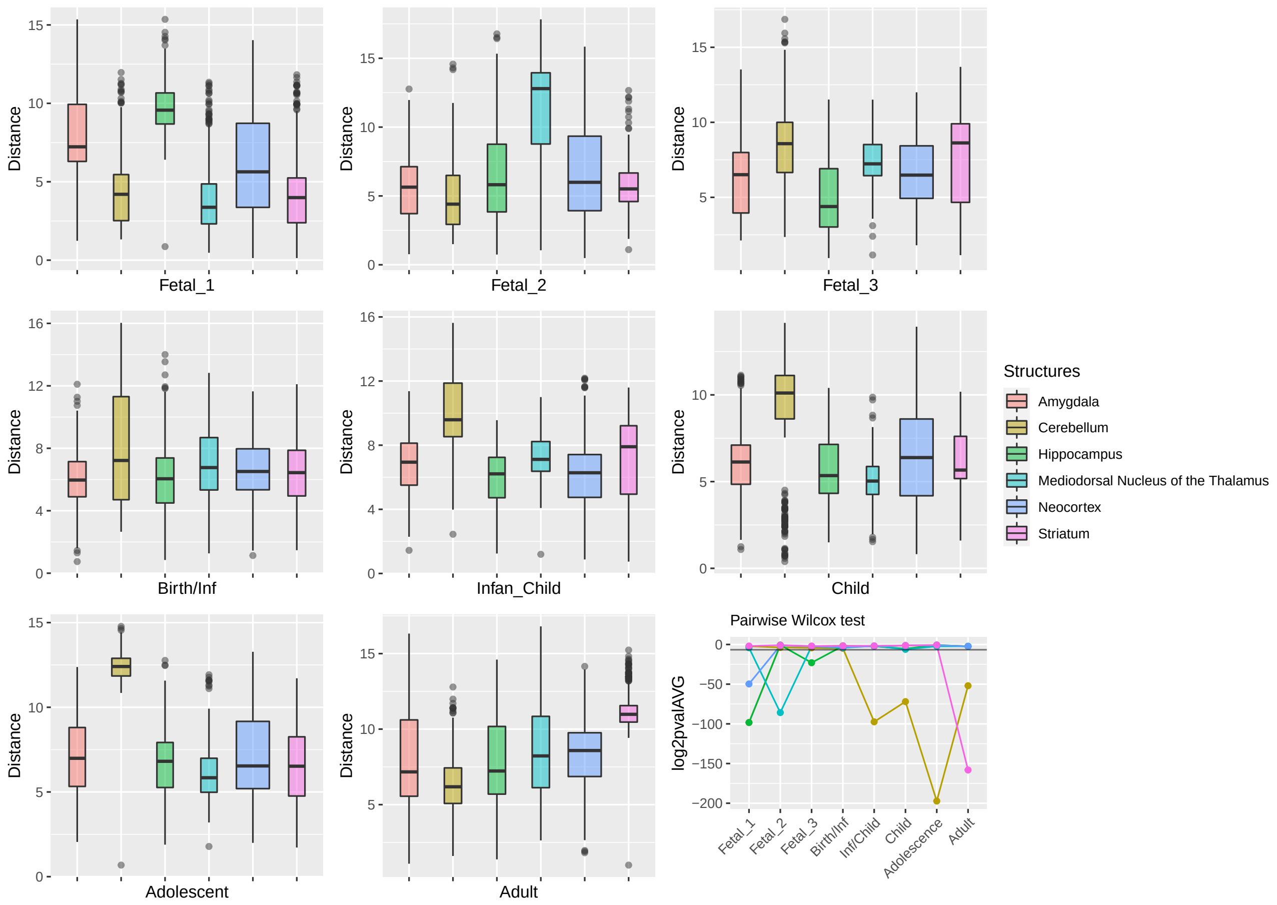

Figure 8: **Evaluation of transcriptomic divergence considering genes within deserts for chromosome 8.** Regarding genes within chromosome 8 desert (n = 46), the cerebellar profile, at successive stages postnatally, stand out as the most divergent transcriptome. The striatum at adulthood and several structures prenatally (neocortex, hippocampus, mediodorsal nucleus of the thalamus) were also statistically different when considering their transcriptomic profiles. All p-values can be found at the online repository `https://github.com/jjaa-mp/desertsHomo`

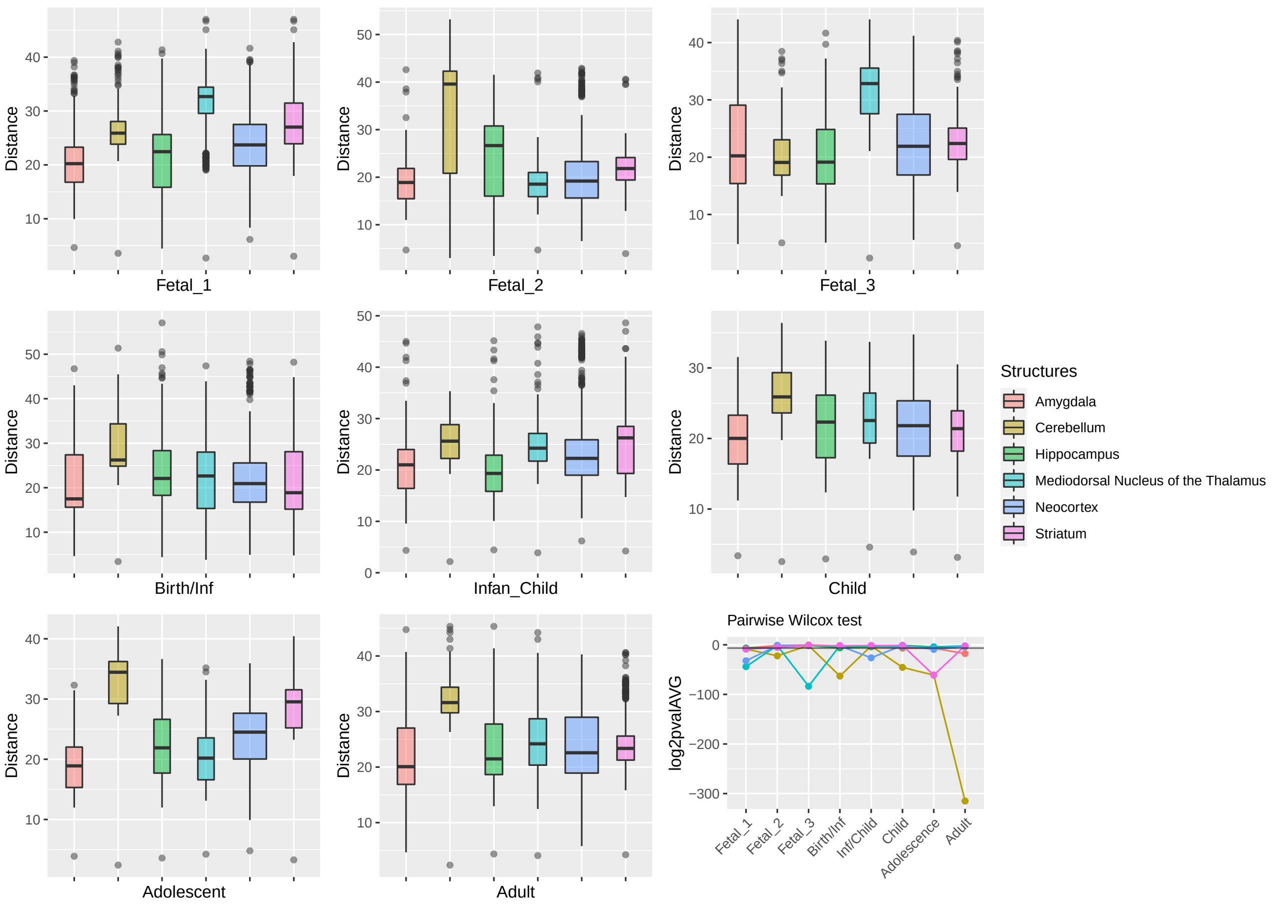

Figure 9: **Evaluation of transcriptomic divergence considering genes under positive selection not within large deserts of introgression.** Similarly to what is found in Supplementary Figure4 and 5, the cerebellum stand out postnatally (birth $p = 1.07\text{x}10^{-19}$, childhood $p = 1.95\text{x}10^{-14}$, adolescence $p = 4.11\text{x}10^{-19}$ and adulthood $p = 1.42\text{x}10^{-95}$). Other significant results are found for the thalamus (fetal stages 1 and 3). striatum (adolescence) or neocortex (fetal stage 1).

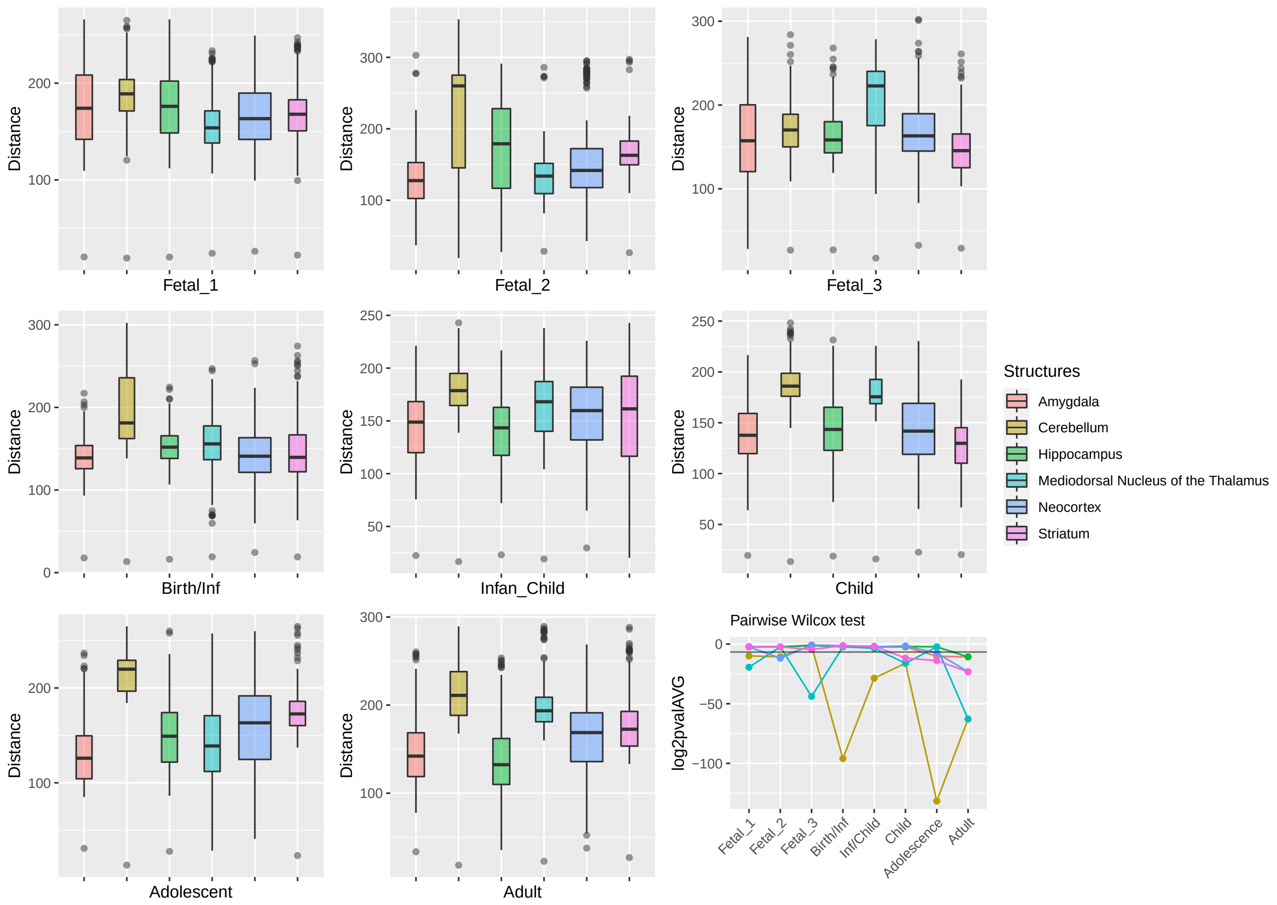

Figure 10: **Global profile of genes across stages and structures.** We processed the mRNA-seq data from [1] filtering out genes with a median across stages and structures less than 2, resulting in a total of 9358 genes. As described in section 4 of the main text, log-transformed, RPKM normalized counts were used to calculate pairwise Euclidean distances using statistically significant principal components for each stage. The most significant differences (pairwise Wilcoxon test with Bonferroni correction) are found for the cerebellum at postnatal stages (birth $p = 1.29\text{x}10^{-29}$; infancy $p = 2.53\text{x}10^{-9}$; adolescence $p = 2.44\text{x}10^{-30}$; adulthood $p = 1.21\text{x}10^{-19}$), and for the thalamus at fetal stage 3 ($p = 6.12\text{x}10^{-14}$) and adulthood ($p = 1.21\text{x}10^{-19}$) . All p-values can be found in Supplementary Material. The horizontal black line in the line plot denotes $p = 0.01$.

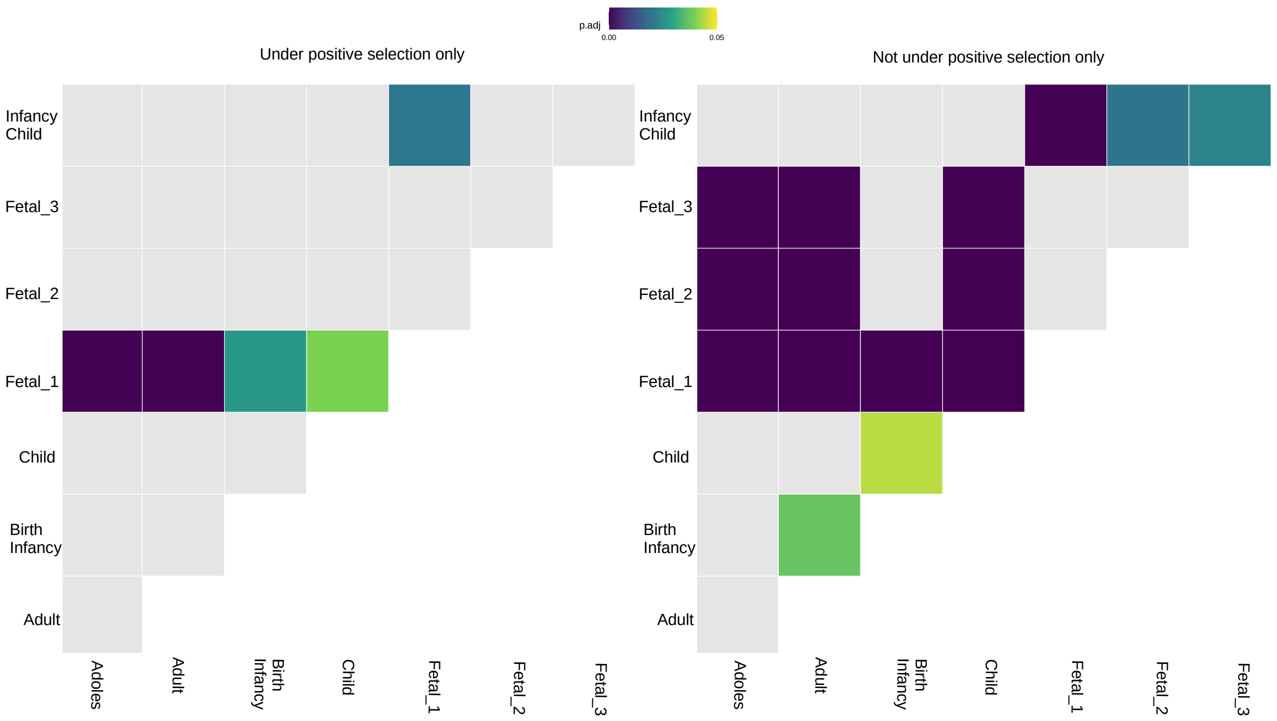

Figure 11: **Visualization of a pairwise post-hoc Tukey test** comparing the mean expression of genes in deserts of introgression under the effect of positive selection (left) and in genes not affected by positive selection (right) in each of the developmental stages included in [1].

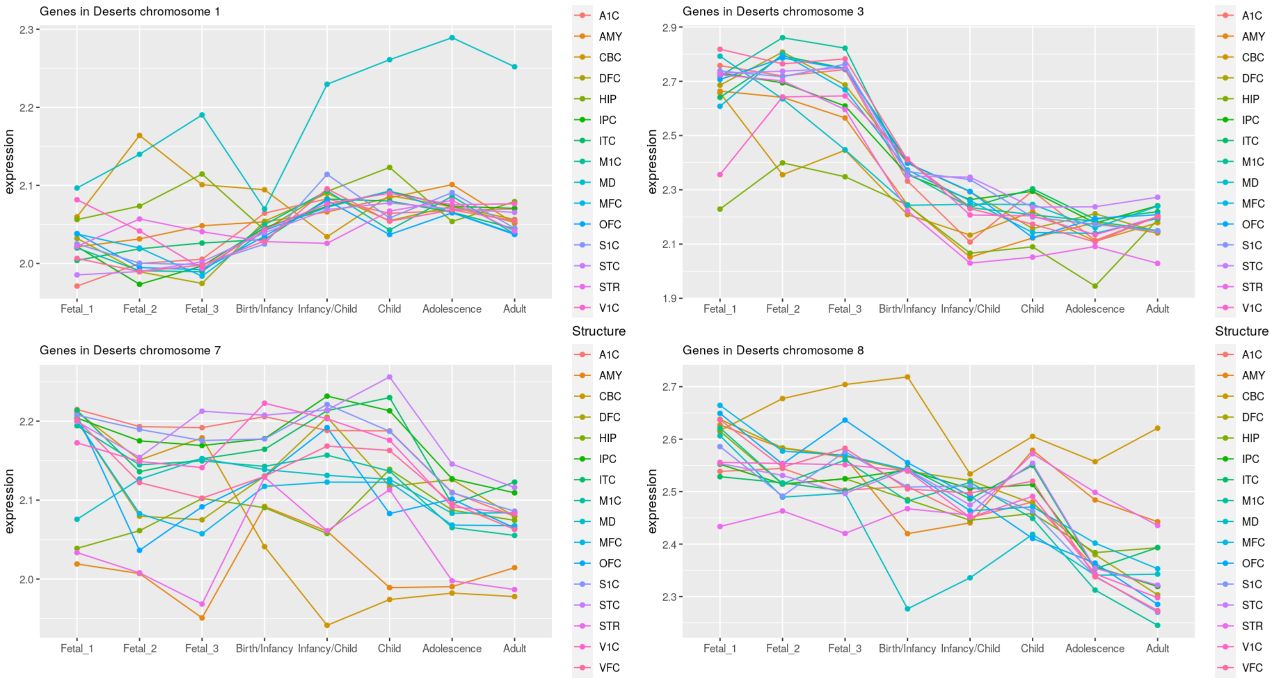

Figure 12: **Decomposition of median expression profile of genes within large deserts per chromosome.** Genes within chromosomes 3 (n = 15) and 8 (n = 46) report higher mean expression values than those within chromosomes 1 (n = 132) and 7 (n = 62), markedly at prenatal stages (as discussed in *Methods* section 4, we kept for these analyses possible outliers, including therefore those with low expression values, which are more abundant in datasets with a higher n. This reinforces the strategies to characterize gene-specific expression dynamics undertaken in this study). Some structures show specific profiles, as the for mediodorsal nucleus of thalamus genes within chromosome 1, or the cerebellum in chromosomes 7 and 8. This view complements Supplementary Figure 13, dedicated to genes under positive selection within introgression deserts.
A1C, primary auditory cortex; AMY, amygdala; CBC, cerebellar cortex; DFC, dorsolateral prefrontal cortex; HIP, hippocampus; IPC, posterior inferior parietal cortex; ITC, inferior temporal cortex; M1C, primary motor cortex; MD, mediodorsal nucleus of thalamus; MFC, medial prefrontal cortex; OFC, orbital prefrontal cortex; S1C, primary somatosensory cortex; STC, superior temporal cortex; STR, striatum; V1C, primary visual cortex; VFC, ventrolateral prefrontal cortex.

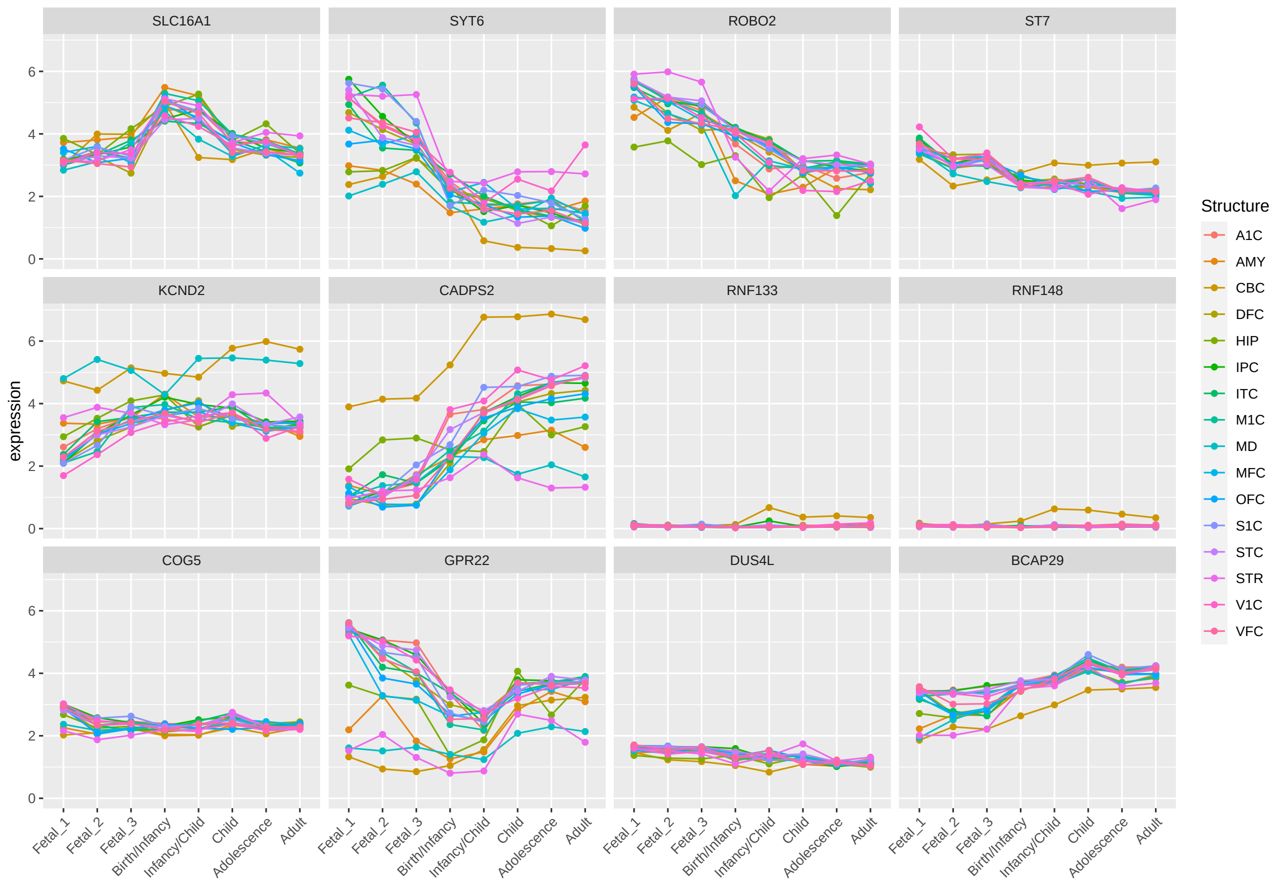

Figure 13: **Expression profile of genes within large deserts and positively-selected overlapping regions.** Twelve protein-coding genes were found at the intersection between large deserts of introgression and putative positively-selected regions. The expression of *CADPS2* and *KCND2* largely recapitulate the unique profile shown before (Supplementary Figure S1) for the cerebellar cortex: Increasing expression from prenatal to postnatal stages, reaching the highest median expression value from childhood to adulthood in comparison to all other structures.
A1C, primary auditory cortex; AMY, amygdala; CBC, cerebellar cortex; DFC, dorsolateral prefrontal cortex; HIP, hippocampus; IPC, posterior inferior parietal cortex; ITC, inferior temporal cortex; M1C, primary motor cortex; MD, mediodorsal nucleus of thalamus; MFC, medial prefrontal cortex; OFC, orbital prefrontal cortex; S1C, primary somatosensory cortex; STC, superior temporal cortex; STR, striatum; V1C, primary visual cortex; VFC, ventrolateral prefrontal cortex.

References

[1] M. Li, *et al.*, "Integrative functional genomic analysis of human brain development and neuropsychiatric risks," *Science*, vol. 362, Dec. 2018.

[2] S. Grote, *et al.*, "ABAEnrichment: an R package to test for gene set expression enrichment in the adult and developing human brain," *Bioinformatics*, vol. 32, pp. 3201–3203, Oct. 2016.

[3] M. Kuhlwilm *et al.*, "A catalog of single nucleotide changes distinguishing modern humans from archaic hominins," *Scientific Reports*, vol. 9, p. 8463, June 2019.

[4] A. Andirkó *et al.*, "Modern human alleles differentially regulate gene expression across brain regions: implications for brain evolution," *bioRxiv*, p. 771816, Nov. 2020. Publisher: Cold Spring Harbor Laboratory Section: New Results.
